# Long-Term Sucralose Consumption Alters Gut Microbiota and Hepatic Lipid Metabolism and Increases Depressive-like Behavior in Young Mice

**DOI:** 10.3390/nu18203452

**Published:** 2026-10-21

**Authors:** Beulah Favour Ortutu, Kyeong-sook Keum, Jin Gyeom Kim, Su-In Yoon, Jin Ah Cho

**Affiliations:** 1 Department of Food and Nutrition, Chungnam National University, Daejeon 34134, Republic of Korea; beulah@tamu.edu (B.F.O.); sd2143@naver.com (K.-s.K.); iigla2005@o.cnu.ac.kr (J.G.K.); 2 Glocal Lifecare Convergence Program, Interdisciplinary Education Center for the Innovative Next Generation Leaders in Glocal Lifecare, Chungnam National University, Daehak-ro 99, Yuseong-gu, Daejeon 34134, Republic of Korea; 3 Research Center for Microbiome-Brain Disorders, Chungnam National University, Daejeon 34134, Republic of Korea; suinyoon@cnu.ac.kr

**Keywords:** sucralose, liver inflammation, gut–liver–brain axis, metabolic dysfunction, neuroinflammation, depressive-like behavior, endoplasmic reticulum stress, intestinal barrier integrity, unpredictable chronic mild stress

## Abstract

**Background**: Sucralose is widely used as a non-nutritive sweetener, yet its systemic consequences of long-term exposure remain incompletely understood. This study investigated the effects of long-term sucralose consumption on metabolic homeostasis and neurobehavioral outcomes. **Methods**: Male C57BL/6J mice were treated with 5 mg/kg body weight sucralose under non-stressed and unpredictable chronic mild stress conditions for 14 weeks. **Results**: Sucralose altered hepatic cholesterol regulation without significantly increasing serum triglyceride or total cholesterol relative to water controls. Significant treatment–stress interactions were detected for hepatic *Srb1* (*F* = 6.511, *p* = 0.004) and intestinal *Npc1l1* (*F* = 3.763, *p* = 0.032), indicating stress-dependent modulation of cholesterol absorption. Hepatic inflammation also showed significant interactions for *Il6* (*p* < 0.001) and *Tnfa* (*p* = 0.003). In addition, the sucralose group exhibited heightened neuroinflammation, cerebral endoplasmic reticulum stress, and increased depressive-like behaviors. 16S rRNA sequencing demonstrated reduced α-diversity and a shift in microbial composition and predicted carbohydrate processing and transport functions. Spearman correlation analysis linked microbial, intestinal, hepatic, neuroinflammatory, and behavioral variables within sucralose-treated mice. **Conclusions**: Long-term sucralose consumption altered gut microbial composition, hepatic cholesterol regulation, and neuroinflammatory and behavioral outcomes.

## 1. Introduction

Due to rising obesity rates and concerns about metabolic disorders such as type 2 diabetes, artificial sweeteners have gained global acceptance as healthier alternatives to sugar [1]. Among the most commonly consumed artificial sweeteners, sucralose (1,6-dichloro-1,6-dideoxy-β-D-fructofuranosyl-4-chloro-4-deoxy-α-D-galactopyranoside) is widely used because of its high sweetness and thermal stability [2,3,4]. The U.S. Food and Drug Administration has established an acceptable daily intake (ADI) of 5 mg/kg of body weight for sucralose [5], while the European Food Safety Authority (EFSA) recently reaffirmed an ADI of 15 mg/kg body weight/day for currently authorized uses [6]. The EFSA concluded that estimated consumer exposure under currently authorized uses remains below this ADI and does not raise a safety concern, while noting uncertainties for certain proposed extensions of use involving prolonged high-temperature processing [6]. These differing regulatory benchmarks, together with emerging mechanistic findings, underscore the importance of continued long-term toxicological and intervention research without implying that currently authorized uses are unsafe.

Sucralose has been widely marketed as safe [7]. However, emerging preclinical, clinical, and epidemiological studies have revealed conflicting results concerning the metabolic effects of sucralose consumption [8]. Some studies have suggested that sucralose consumption can promote adiposity, insulin resistance, glucose intolerance, and weight gain [9,10,11], while others report minimal or no adverse metabolic impacts [12,13,14]. Although these discrepancies may result from differences in study design, dosage, or health status among participants and experimental animals, they have contributed to uncertainty about the long-term health consequences of sucralose use.

In addition, evidence indicates that sucralose may have biological effects extending beyond metabolism [7]. Preclinical studies have reported alterations in gut microbiota composition following exposure to sucralose and other non-nutritive sweeteners [15,16,17]. In experimental colitis and colitis-associated colorectal cancer models, sucralose has also been shown to aggravate intestinal barrier dysfunction and inflammatory signaling [18,19]. Studies have also linked sucralose consumption to liver dysfunction and the development of non-alcoholic fatty liver disease (NAFLD) [20,21,22]. Additionally, impaired immune responses, including reduced T-cell differentiation, have been reported [23]. Taken together, these observations suggest that sucralose might disrupt the gut–liver axis, which plays a central role in coordinating metabolic and immune functions. In turn, chronic sucralose consumption could contribute to neurobiological dysfunction.

The gut–liver–brain axis represents an integrated communication network through which intestinal microbiota composition and barrier integrity regulate hepatic metabolism, immune signaling, and neurobehavioral outcomes [24]. Disruption of the intestinal barrier has been shown to increase the delivery of inflammatory mediators to the liver through the portal circulation, promoting hepatic inflammation and metabolic dysregulation. In turn, liver-derived cytokines, lipid metabolites, and bile acids can influence central nervous system function either directly or indirectly through the liver–brain axis [25]. However, despite these findings, few studies have comprehensively examined how long-term sucralose consumption affects metabolic, inflammatory, and neurobehavioral outcomes simultaneously. Addressing this knowledge gap is important for clarifying the health risks associated with sucralose intake.

In addition, most studies fail to consider the impact of chronic stress as a confounding factor when assessing sucralose’s health effects. This oversight is significant because chronic stress is ubiquitous in modern life and substantially modifies physiological responses to dietary interventions. Preclinical and clinical studies have linked chronic stress to increased vulnerability to metabolic disorders, inflammation, and neurological disorders, such as depression and anxiety [26,27,28]. At the cellular level, chronic stress in daily life has been reported to trigger cellular stress responses [29]. Chronic stress and proinflammatory cytokines can impair protein folding in the endoplasmic reticulum (ER), leading to ER stress [30]. ER stress triggers the unfolded protein response (UPR) through PKR-like ER kinase (PERK), activating transcription factor 6 (ATF6), and inositol-requiring enzyme 1 (IRE1) to maintain ER homeostasis [31]. The downstream effects of ER stress, including inflammation and the generation of reactive oxygen species (ROS), contribute to oxidative stress, which is a primary cause of disrupted cellular homeostasis [28]. Chronic stress may act as a physiological amplifier of diet-induced inflammatory signaling, ER stress, and oxidative imbalance, thereby, increasing susceptibility to sucralose-induced metabolic and neurobehavioral dysfunction.

Despite the widespread use of sucralose, uncertainty remains regarding how sustained exposure in the young age may influence metabolic, intestinal, inflammatory, and neurobehavioral outcomes under chronic stress. To address these gaps, our study evaluated these outcomes concurrently under non-stressed and chronic stress conditions by integrating physiological, molecular, and behavioral assessments across multiple organ systems.

## 2. Materials and Methods

### 2.1. Animals

Three-week-old male C57BL/6J mice were purchased from DBL Laboratory Animal Co., Ltd. (Eumseong, Republic of Korea). Upon arrival, the mice underwent 1-week quarantine period. Four-week-old mice were selected to capture early-life exposure to sucralose during a developmental window relevant to gut microbiota establishment and neurobehavioral maturation [32,33]. Mice were housed under controlled conditions at constant humidity (55 ± 10%) and temperature (24.0 ± 2.0 °C) with a 12 h light/dark cycle (lights on at 7:00 a.m.). A chow diet, consisting of 18.4% protein, 44.2% carbohydrate, 3.8% crude fiber, and 6% fat (2018S/2018SC, Inotiv, Madison, WI, USA), together with water, was provided ad libitum.

The mice were euthanized with CO_2_ at the end of all the experiments. Blood, brain, epididymal white adipose tissue (eWAT), subcutaneous white adipose tissue (sWAT), mesenteric white adipose tissue (mWAT), perirenal white adipose tissue (PRAT), brown adipose tissue (BAT), liver, small intestine (SI), and colon tissues were harvested and prepared for analysis accordingly [34]. eWAT was selected for histological assessment as a representative visceral white adipose depot in male mice because visceral adipose tissue is particularly responsive to diet-related hypertrophy and inflammatory remodeling; sWAT was analyzed in parallel to evaluate depot-specific gene expression responses.

The liver, SI, colon, and fat tissues were weighed and thoroughly rinsed in ice-cold PBS (Corning, NY, USA) immediately after being harvested to reduce residual red blood cell contamination. All animal experiments were approved by the Animal Experiment Ethics Committee of Chungnam National University, Daejeon, Republic of Korea (202304A-CNU-074).

### 2.2. Experimental Design

#### 2.2.1. Sucralose Treatment

Forty-eight mice were randomly assigned to six groups (*n* = 8 per group). Treatments were dissolved in drinking water and administered ad libitum starting at four weeks of age and continuing until the end of the experiment. The three treatment conditions were as follows: water (W) as a negative control; 30% *w*/*v* sucrose (S; DUKSAN Pure Chemical Co., Ltd., Ansan, Republic of Korea) as a high-calorie comparative control; and 5 mg/kg body weight sucralose (US; ES food, Gunpo, Republic of Korea) as the experimental group. The 30% (*w*/*v*) sucrose concentration was selected to provide a high-calorie comparator, consistent with a previous study [35]. The sucralose dose was selected to correspond to the US FDA ADI without implying equivalent biological exposure across species. The sucrose and sucralose solutions were not matched for sweetness, as the experimental conditions were selected on the basis of sucrose concentration and sucralose dose rather than equivalent sweetness. Groups were further divided into non-stressed groups (W, S, US) and groups exposed to unpredictable chronic mild stress (UCMS; W+, S+, US+, respectively). The 14-week exposure period was selected to model sustained exposure from adolescence into young adulthood and to allow chronic metabolic, microbiome, and stress-related phenotypes to develop; it was not intended to reproduce a specific human consumption pattern. Throughout the study, food intake was measured weekly, accounting for spillage, while fluid intake was recorded every other day by weighing the bottles. Caloric intake was calculated based on the energy density of the standard chow (3.1 kcal/g) and supplemental calories from the sucrose solutions. Body weight was recorded weekly using a calibrated digital scale.

#### 2.2.2. UCMS Treatment

The UCMS model was employed using established procedures to induce chronic stress (Table S1) [36]. Stressors were applied throughout the 14-week experimental period according to the schedule summarized in Table S1. Stressors included: cage tilting (45°) for 24 h; wet bedding (250–500 mL water) for 24 h; food or water deprivation for 24 h; forced swimming (21 °C or 28 °C) for 10 min; light/dark cycle reversal; physical restriction in a 50 mL tube (Hyundai Micro, Anseong, Republic of Korea) for 2 h; tail pinching for 5 min using sterilized forceps; and shaking the cage at 150 rpm for 10 min. Additionally, mice were individually kept in an empty cage without bedding or nesting material for 24 h [36,37]. To prevent transmission of stress-related olfactory or auditory cues to control groups, each experimental group (*n* = 8, 4 mice per cage) was housed in separate racks in different areas of the facility. Mice were returned to their designated stress conditions immediately following behavioral tests. Dietary treatment and chronic stress exposure were administered simultaneously from 4 to 18 weeks of age.

### 2.3. Assessment of Depressive-like Behavior

The forced swim test (FST) assessing depressive-like behavior was performed when the mice were 15 weeks old [38]. The test was performed in a transparent cylinder (30 cm high × 20 cm diameter) filled with approximately 15 cm of water at 23 to 25 °C. Mouse behavior was video recorded for 6 min using PanLab SMART Video Tracking software 3.0 (Harvard Apparatus, Holliston, MA, USA). Since most mice attempted to escape in the first 2 min, immobility time was measured over the final 4 min.

### 2.4. Histopathological Analysis

Portions of the liver, eWAT, ileum, and colon were fixed in 10% neutral buffered formalin (BBC Biochemical, Mount Vernon, WA, USA) for hematoxylin and eosin (H&E) staining as described by Feldman and Wolfe [39]. Adipocyte area and the number of adipocytes per unit area in eWAT, epithelial damage, and inflammatory cell infiltration in the colon and ileum, and non-alcoholic fatty liver disease (NAFLD) activity in liver tissues were analyzed. The NAFLD activity score combined inflammation, steatosis, and ballooning, were analyzed [40]. A score of 0 was assigned to indicate the absence of NAFLD, while a score of 8 was used to indicate severe NAFLD. All evaluations were conducted by three different research assistants to enhance the reliability of the results. A double-blind approach was employed, whereby the sample names and numbers were concealed from the research assistants and the researcher to avoid unconscious bias. An optical microscope (BHS-312; Olympus Optical Co., Ltd., Tokyo, Japan) was used at magnifications of 4×, 10×, and 40×. Adipocyte size was assessed using ImageJ software (version 1.54p, NIH, Bethesda, MD, USA).

### 2.5. Biochemical Analysis

#### 2.5.1. Glucose Metabolism Assessment

Blood was collected through cardiac puncture using a 1 mL syringe after a 12 h fasting period at the experimental endpoint. Fasting blood glucose was measured using a One Touch Ultra glucometer (LifeScan, Malvern, PA, USA), and serum insulin levels were assessed using an Ultra-Sensitive Mouse Insulin ELISA Kit (Crystal Chemicals Co., Ltd., IL, USA). Insulin resistance was then estimated using the homeostatic model assessment for insulin resistance (HOMA-IR) using the following formula:

$$\text{HOMA-IR}=\frac{\mathrm{Fasting}\;\mathrm{insulin}\;(µU/\mathrm{mL})\times \mathrm{Fasting}\;\mathrm{glucose}\;(\mathrm{mg}/\mathrm{dL})}{22.5}$$

#### 2.5.2. Lipid Profile Analysis

Serum and hepatic total cholesterol (TC) and triglyceride (TG) levels were measured using Manual TC (Cat #150T) and Manual TG (Cat #110T) kits, respectively (Asan Pharmaceutical Co., Ltd., Seoul, Republic of Korea) following the manufacturer’s instructions. The absorbance was measured within 60 min at 500 nm for TC and 550 nm for TG using a Microplate Spectrophotometer (Thermo Fisher Scientific, Waltham, MA, USA).

Total lipids in liver tissue were extracted according to the methods of Bligh and Dyer [41]. Approximately 3 mL of CHCl_3_/methanol (1:2) containing 0.01% butylated hydroxytoluene solution was added to 1 g of liver tissue, which was subsequently homogenized using an MP homogenizer (MP Biomdeicals, Irvine, CA, USA) at 900 rpm for 5 min at 4 °C. After homogenization, 3 mL of 2 M KCl with 0.1 M EDTA solution was added and was mixed by pipetting and centrifuged at 37 °C for 30 min. Another 3 mL of 1N CHCl_3_ was added and mixed under the same conditions. The same process was repeated twice, followed by the collection of 200 µL of the supernatant for lipid profile analysis.

### 2.6. Molecular Analysis

#### 2.6.1. RNA Extraction, cDNA Synthesis, and Polymerase Chain Reaction (PCR)

Total RNA from brain, liver, colon, SI, and adipose tissues was extracted with TRI reagent (Molecular Research Center Inc., MRC, Cincinnati, OH, USA) using a GentleMACS™ Dissociator (Miltenyi Biotec Co., Bergisch Gladbach, Germany). The RNA concentration was measured using a Nanodrop spectrophotometer (Thermo Fisher Scientific Inc., Waltham, MA, USA), and cDNA was synthesized using an RT-kit (BioFACT Co., Daejeon, Republic of Korea).

Quantitative real-time PCR (qRT-PCR) was conducted using 2× real-time PCR Master Mix containing SYBR Green (BioFACT Co, Daejeon, Republic of Korea) on an AriaMX Real-time PCR system (Agilent Co., Santa Clara, CA, USA). Reverse Transcription PCR (RT-PCR) was conducted using 2× Taq Basic PCR Master Mix 2 (BioFACT Co, Daejeon, Republic of Korea). PCR samples were loaded and run on a 1.5% agarose gel (for *Pparγ*) and a 2% agarose gel (for *Fas* and *Gapdh*). Gel images were captured under UV light using an AE-9000 E-graph (ATTO, Tokyo, Japan).

#### 2.6.2. Western Blotting

Liver tissue was homogenized in RIPA lysis buffer (Thermo Fisher Scientific Inc., Waltham, MA, USA) with phosphatase inhibitor (Roche, Basel, Switzerland) and protease inhibitor cocktail (Thermo Fisher Scientific Inc., Waltham, MA, USA) using a GentleMACS™ Dissociator (Miltenyi Biotec Co., Bergisch Gladbach, Germany), and the protein concentrations were measured using a bicinchoninic acid (BCA) protein assay kit (Thermo Fisher Scientific Inc., Waltham, MA, USA). Protein lysates were run on 10% polyacrylamide gels and transferred to nitrocellulose membranes. The membranes were then blocked with 5% skim milk in Tris-buffered saline containing Tween 20 (TTBS) and incubated overnight at 4 °C with primary antibodies. The primary antibodies used were against FXR/NR1H4 (Cat #72105, Cell Signaling Technology, Danvers, MA, USA, 1:1000) and Vinculin (Cat #SC-73614, Santa Cruz Biotechnology, Inc., Texas, LA, USA, 1:1000). The membranes were then washed with 1× TTBS three times, incubated with secondary goat anti-rabbit antibody (Invitrogen, Waltham, MA, USA, 1:10,000) for 1 h, and washed with 1× TTBS three times. Visualization was performed with chemiluminescent Western blot reagents (Thermo Fisher Scientific Inc., Waltham, MA, USA.) and a ChemiDoc system (ATTO Co., Tokyo, Japan).

### 2.7. 16S rRNA Gene Amplicon Sequencing

Genomic DNA was extracted from mouse fecal samples for 16S rRNA gene amplification sequencing using the DNAeasy PowerSoil Pro Kit (QIAGEN, Germantown, MD, USA) according to the manufacturer’s protocol. The sequences of primers used were 341F (5′-CCTACGGGNGGCWGCAG-3′) and 785R (5′-GACTACHVGGGTATCTAATCC-3′), which target the V3–V4 region of the 16S rRNA gene. The PCR mixture was incubated at 94 °C for 3 min, followed by 25 cycles of 94 °C for 45 s, 51 °C for 1 min, and 72 °C for 10 min. After the amplification products were purified, the amplicons were cleaned and indexed using the Illumina MiSeq Sequencing platform (Illumina, San Diego, CA, USA). DADA2 was used to demultiplex, quality filter, trim and denoise the reads using the pseudo-pooling method. Reads were truncated according to quality score profiles before downstream taxonomic analysis. The obtained taxonomic assignment data were clustered, and species annotation was conducted and classified on the basis of the SILVA database, with taxonomic assignment performed using QIIME2 Version 2024.5 (https://qiime2.org).

### 2.8. Bioinformatics and Statistical Analysis

Statistical analyses were performed using SPSS version 25.0 for Windows (SPSS IBM Corp., Armonk, NY, USA) and the R statistical package (ver. 4.3.1, Vienna, Austria). All measurements are presented as means ± SD. A two-way ANOVA (3 × 2 factorial design) was performed to evaluate the main effects of treatment and chronic stress, as well as their interaction. When a significant interaction effect was detected, Duncan’s post hoc test was conducted to identify specific group differences. To evaluate the effect of stress within each treatment, Student’s *t*-test was used to compare the non-stressed and chronic stress groups. Differences were considered statistically significant at *p* < 0.05. For microbiota analysis, alpha diversity (Shannon index) was assessed using the Kruskal–Wallis test. Alpha diversity boxplots were generated using R software (ver. 4.3.1, Vienna, Austria). Beta diversity was assessed using weighted Bray–Curtis distance, visualized through principal coordinate analysis (PCoA), and tested using PERMANOVA. Taxonomic assignment was performed using the SILVA 138 database [42]. Spearman rank correlations between gut-, liver-, brain-related markers were computed using the Hmisc package, with a significance threshold of *p* < 0.05. Correlation matrices were visualized as heatmaps using ggplot2 in R. Differentially abundant bacterial families were identified using a linear discriminant analysis effect size (LEfSe) framework. Family-level abundance data were normalized within each sample to a total of 1 × 10^6^. Treatment (W, S, and US) was specified as the main class, whereas stress condition (non-stress and UCMS) was incorporated as a subclass to identify treatment-associated taxa showing a consistent direction of change across stress strata. Features were first screened using the Kruskal–Wallis rank-sum test (α = 0.05), followed by subclass consistency testing. LDA effect sizes were estimated using 30 bootstrap iterations with two-thirds resampling. Bacterial families with a Kruskal–Wallis *p* value < 0.05 and an LDA score > 2.0 were considered discriminative taxa and were included in the LDA score bar plot.

## 3. Results

### 3.1. Physiological Changes in Response to Long-Term Sucralose Consumption

**Figure 1:**
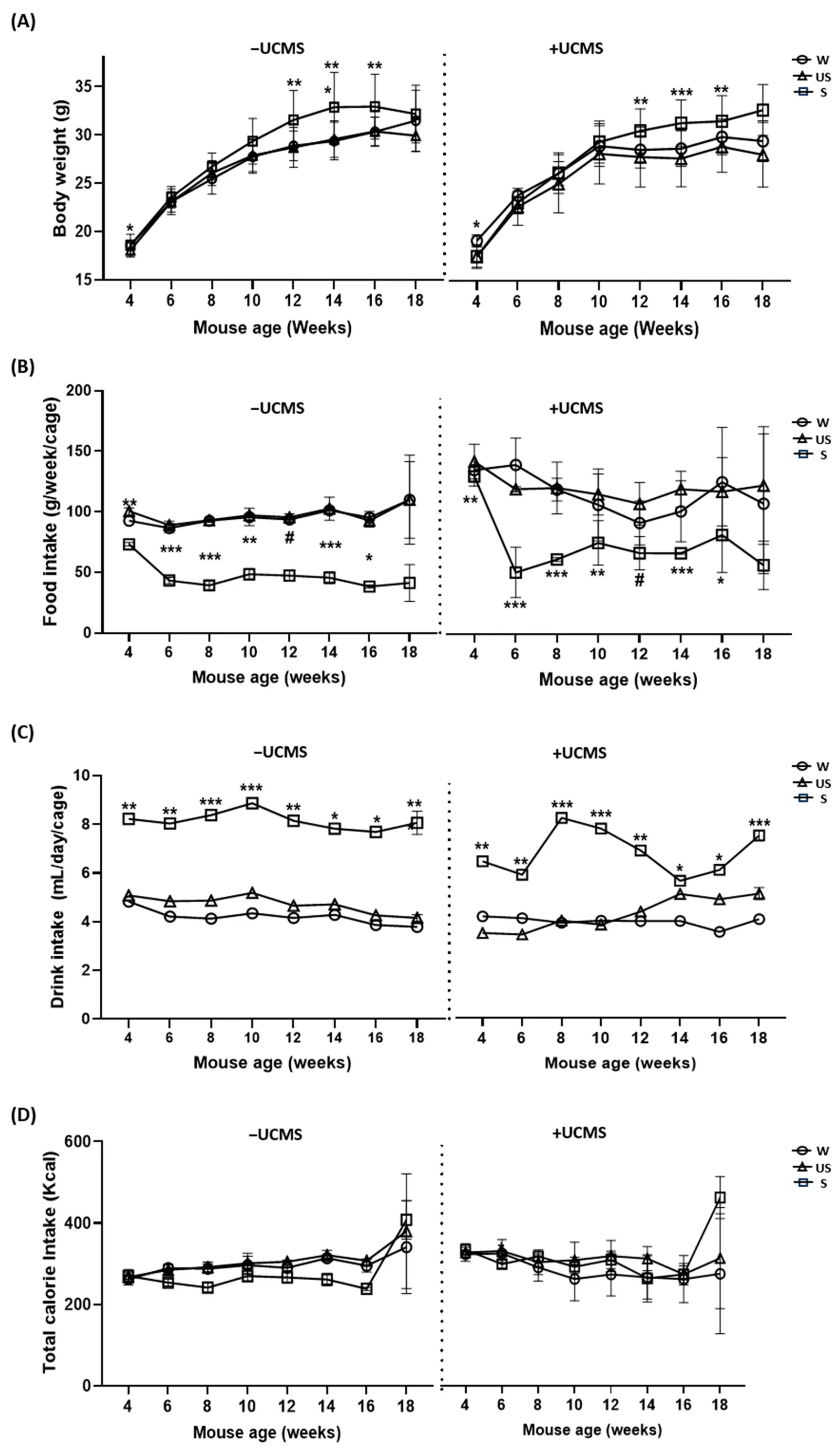

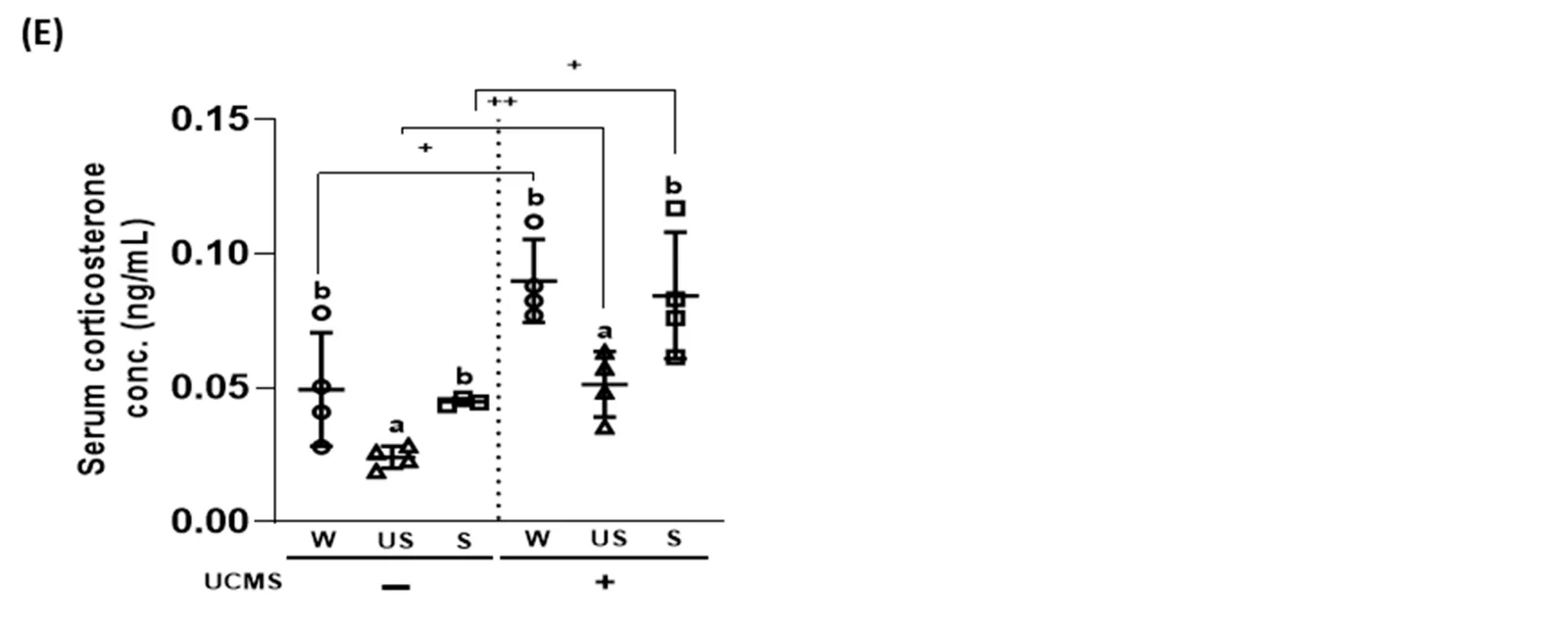
The effects of sucralose, sucrose and chronic stress on body weight, food and fluid intake, total caloric intake, and serum corticosterone. (**A**) Body weight, (**B**) food intake, (**C**) fluid intake, and (**D**) total calorie intake during the experimental period (*n* = 8 per group). (**E**) Serum corticosterone levels at the study endpoint (*n* = 5 per group). Data are presented as means ± SD. Main effects of US treatment were assessed by two-way ANOVA (* *p* < 0.05; ** *p* < 0.01; *** *p* < 0.001), and treatment X UCMS interactions were indicated by # (*p* < 0.05). When no significant interaction was detected by two-way ANOVA, US treatment effects were evaluated using Duncan’s post hoc test; different letters indicate significant differences among treatment groups within the same stress condition (*p* < 0.05). Differences between non-stress and UCMS groups within each treatment were assessed by Student’s *t*-test (^+^ *p* < 0.05; ^++^ *p* < 0.01). W, water; US, sucralose; S, sucrose; UCMS, unpredictable chronic mild stress.

Changes in body weight, as well as food, water, and calorie intake, provide valuable indicators of energy balance and overall metabolic health [43]. At the onset of the study, body weights were comparable across all groups. The S group exhibited significantly greater weight gain under both non-stressed and stressed conditions compared to the other groups (Figure 1A). This was characterized by predictably higher fluid intake (Figure 1C) and simultaneously lower solid food intake (Figure 1B).

At week 12, two-way ANOVA showed a significant interaction between treatment and stress for food intake (*p* = 0.035). The S group consumed less solid food than the other groups, and the magnitude of this reduction was smaller in the stressed S group than in the corresponding non-stressed S group. Because feeding responses to chronic stress can vary with the stress paradigm and dietary context, we interpret this finding as treatment-by-stress interaction rather than as evidence that stress generally increases food intake. Despite these fluctuations, total caloric intake remained largely consistent across groups, except for a transient decrease in the S+ group during week 6 (Figure 1D). The US group maintained body weight, food intake, and fluid intake levels comparable to the W group throughout the experiment under both conditions.

Successful UCMS induction was confirmed by serum corticosterone levels, which were significantly elevated in all stressed groups relative to their non-stressed counterparts (Figure 1E). Interestingly, the US group displayed significantly lower serum corticosterone levels compared with both the W and S groups, regardless of stress conditions.

### 3.2. Long-Term Sucralose Consumption Minimally Affected Adiposity but Induced Depot-Specific Inflammatory Changes in eWAT and sWAT

**Figure 2:**
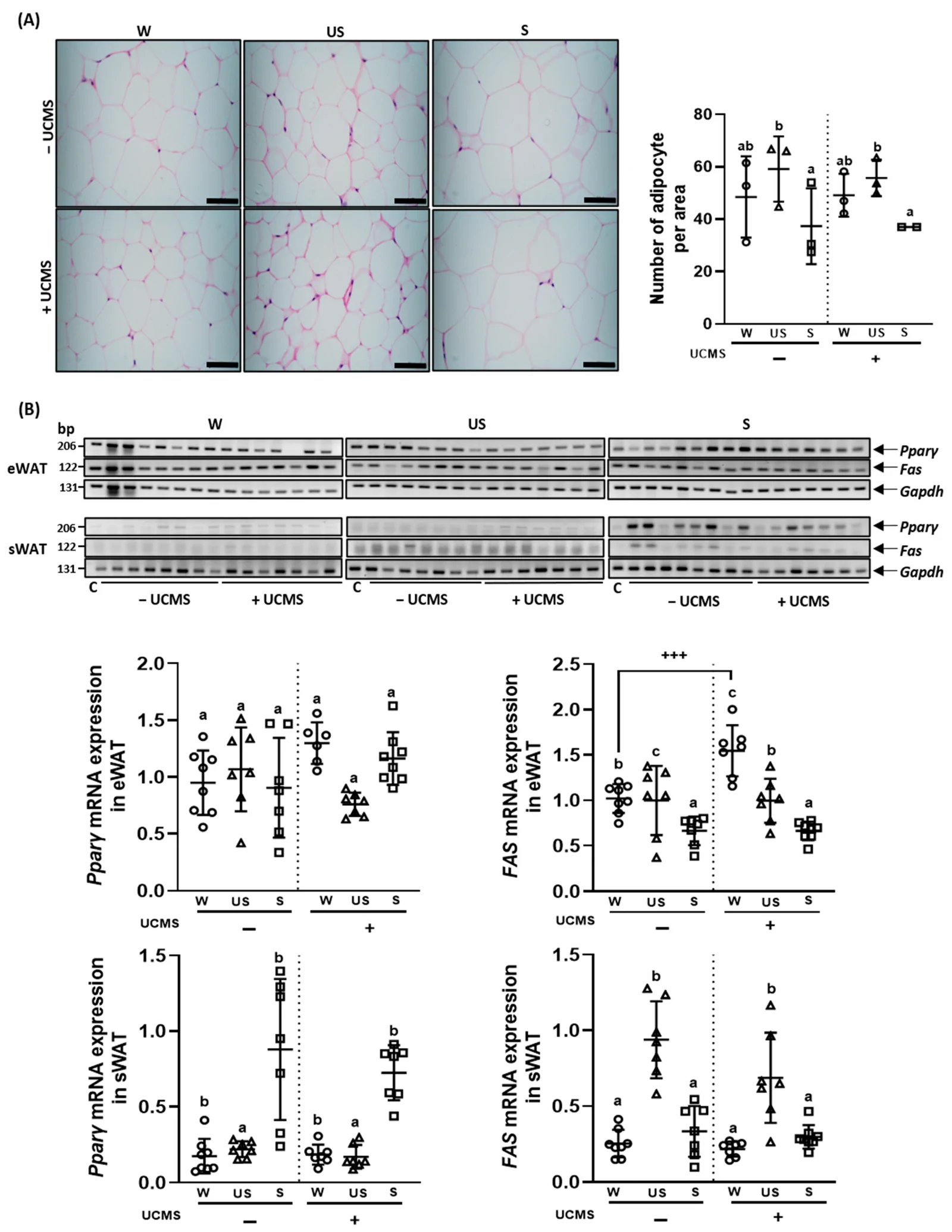

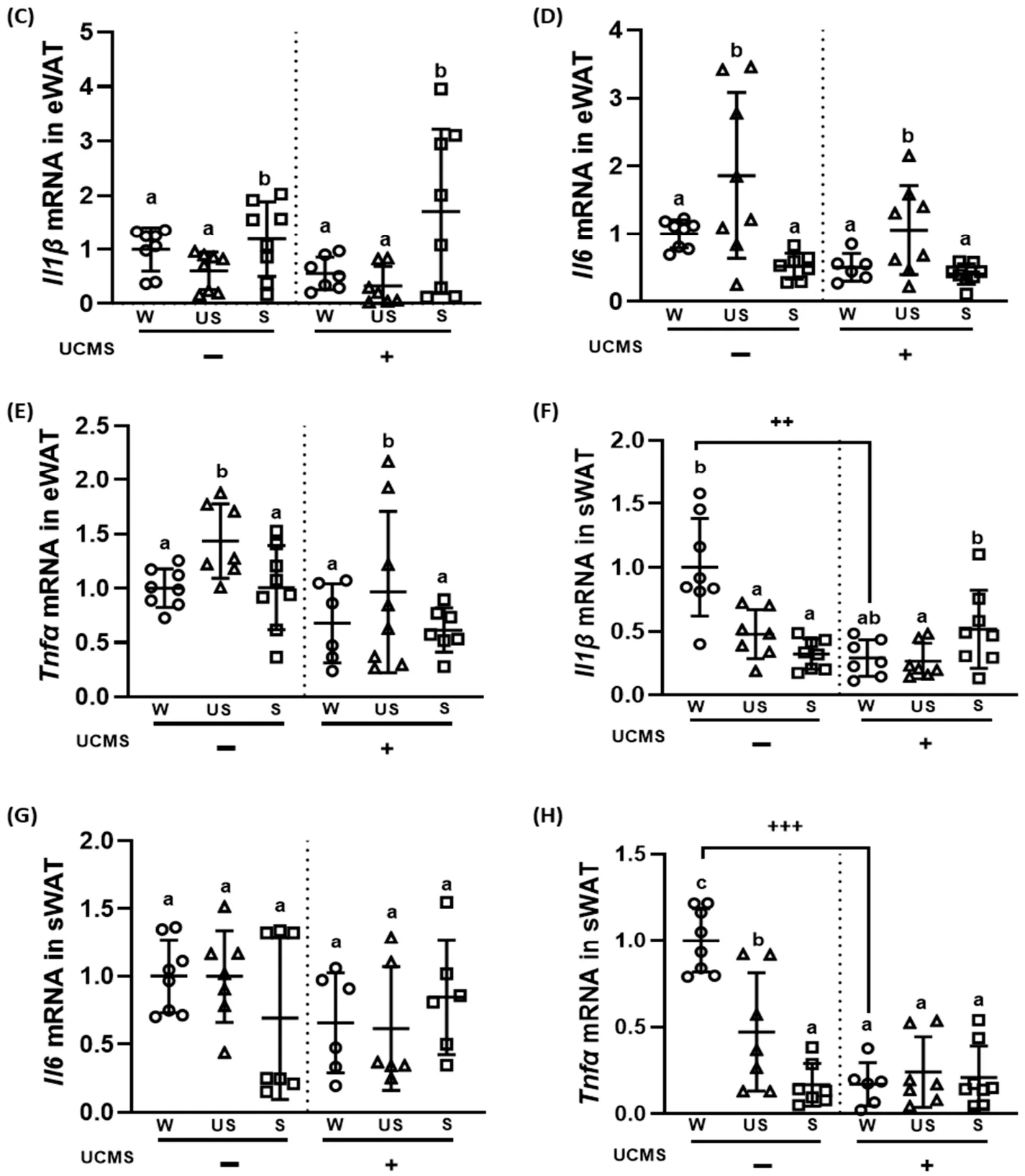
The impact of sucralose and chronic stress on adiposity and depot-specific inflammation. (**A**) Representative H&E-stained images of eWAT (40× magnification; scale bar: 50 µm). Quantification of the number of adipocytes per unit area was performed using ImageJ software. (**B**) *Pparγ* and *Fas* mRNA levels in eWAT and sWAT by RT-PCR and its relative quantification. *Gapdh* as a loading control. Relative mRNA expression of proinflammatory cytokines in eWAT (**C**–**E**) and sWAT (**F**–**H**). Data are presented as means ± SD. When no significant interaction was detected by two-way ANOVA, US treatment effects were evaluated using Duncan’s post hoc test; different letters indicate significant differences among treatment groups within the same stress condition (*p* < 0.05). Differences between non-stressed and UCMS conditions within each treatment were assessed by Student’s *t*-test (^++^ *p* < 0.01; ^+++^ *p* < 0.001). W, water; US, sucralose; S, sucrose; eWAT, epididymal white adipose tissue; sWAT, subcutaneous white adipose tissue; C, intra-control for RT-PCR; UCMS, unpredictable chronic mild stress.

To assess the impact of sucralose on adiposity, we calculated adipose tissue mass relative to total body weight. Total fat percentage was significantly higher in the S group than the W and US groups under both conditions (Figure S1A). Similarly, the measured adipose depots in the S group showed higher weight percentages than those in the W and US groups (Figure S1B–E). eWAT histology showed the most prominent adipocyte hypertrophy in the S group (Figure 2A). Quantification of the number of adipocytes per unit area showed that neither US nor S differed significantly from W; however, the US group had significantly more adipocytes per area than the S group, and chronic stress did not significantly modify this pattern.

Adipogenic and lipogenic markers also showed depot-specific patterns (Figure 2B). In eWAT, Peroxisome proliferator-activated receptor gamma (*Pparγ*) expression did not differ significantly among W, US, and S in both conditions. However, in sWAT, US showed significantly lower *Pparγ* expression than W and S groups.

For fatty acid synthase (*Fas*) in eWAT, the treatment pattern differed by condition, with a significant treatment-by-stress interaction (*F* = 6.203, *p* = 0.005). Under non-stressed condition, US showed higher *Fas* expression than both W and S, whereas under chronic stress condition, W showed the highest expression, US was intermediate, and S remained lowest. In sWAT, both non-stressed and chronic stress conditions, US showed higher *Fas* expression than both W and S.

Overall, these data indicate that sucralose did not produce the adipocyte hypertrophy observed with sucrose, but it was associated with depot-specific changes in adipogenic and lipogenic markers. Because some treatment differences were significant relative to S but not W, these findings are interpreted as differential metabolic remodeling rather than as a uniform increase or decrease in adipogenesis.

Depot-specific inflammatory patterns were also shown. In eWAT, *Il1β* expression in US did not differ from W but was lower than S under both stress conditions (Figure 2C). In contrast, *Il6* and *Tnfα* expression in US were higher than both W and S under non-stressed and stressed conditions (Figure 2D,E).

However, the inflammatory response was uniquely sensitive to chronic stress in sWAT. In sWAT, the inflammatory response was dependent on both treatment and stress. Under non-stressed conditions, *Il1β* expression was lower in US and S than in W; under chronic stress, US was lower than S, while W was intermediate (*F* = 13.855, *p* < 0.001) (Figure 2F). *Il6* did not differ significantly among treatment groups (Figure 2G). For *Tnfα,* W showed the highest expression under non-stressed conditions, US was intermediate, and S was lowest; these between-treatment differences were not retained under chronic stress (Figure 2H). The significant stress-related decrease in W further contributed to the treatment by stress interaction.

In summary, our data showed downregulation of *Pparγ* and upregulation of *Fas* in sWAT suggesting impaired adipocyte differentiation and enhanced lipogenic capacity in the US group. Taken together, the differential responses observed between eWAT and sWAT suggest that chronic sucralose consumption induces adipose depot-specific metabolic and inflammatory remodeling rather than a single directional effect of sucralose.

### 3.3. Long-Term Sucralose Consumption Altered Hepatic Lipid Metabolism and Induced Liver Inflammation

**Figure 3:**
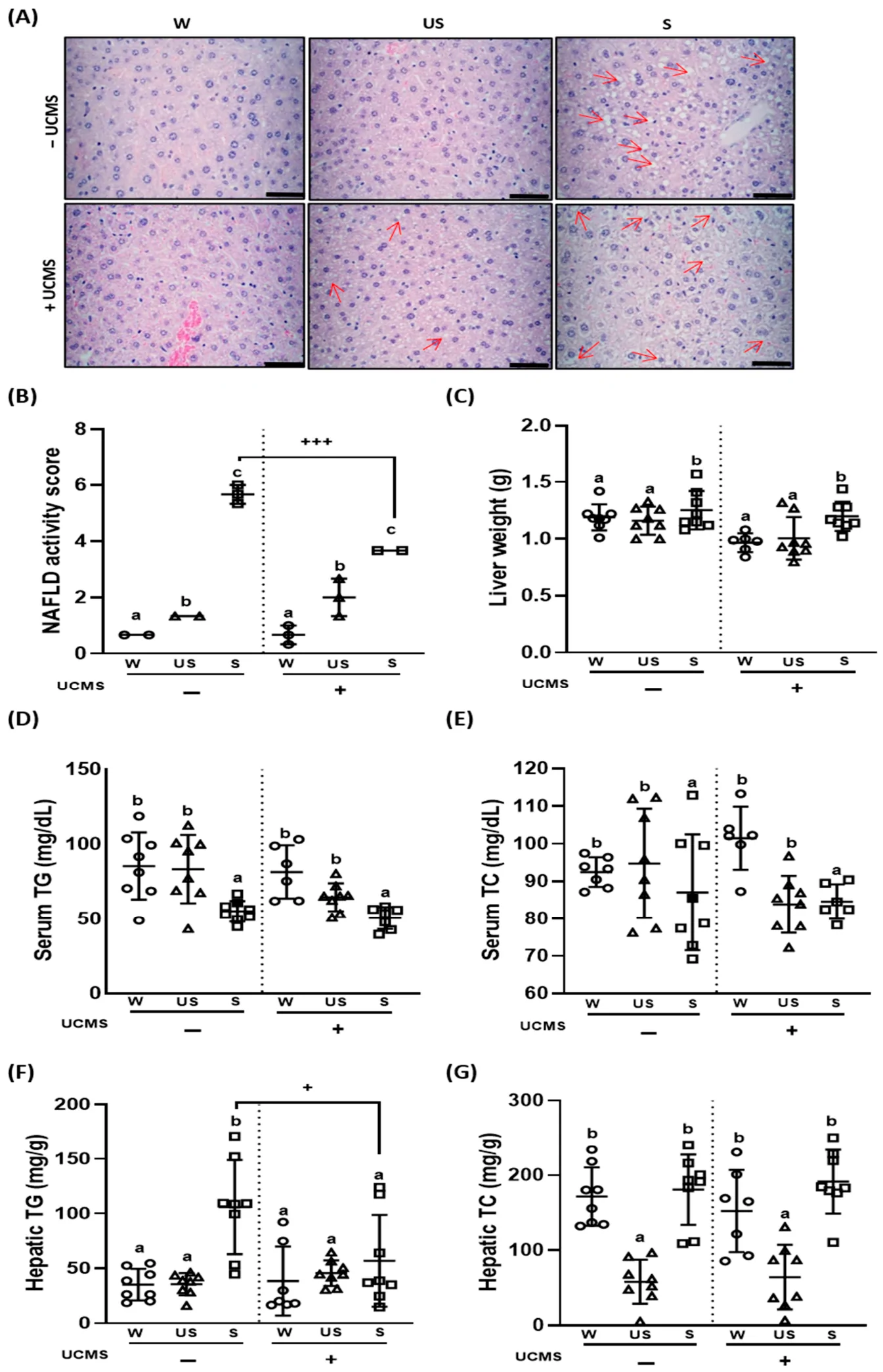

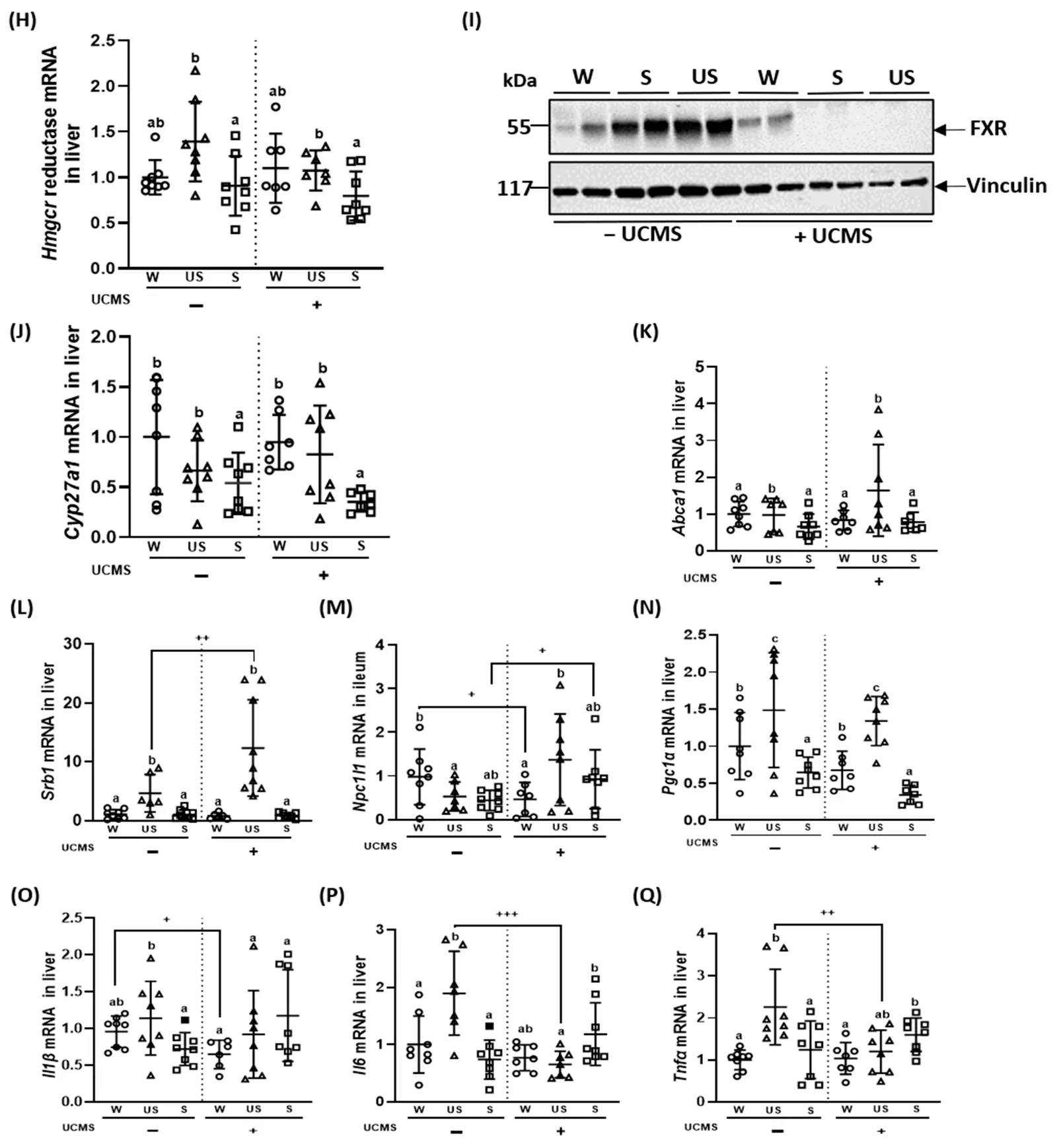
The impact of chronic stress on sucralose-induced hepatic lipid dysregulation and inflammatory response. (**A**) Representative H&E-stained images (40× magnification; scale bar: 50 µm). Red arrows indicate steatosis and ballooning. (**B**) NAFLD activity score and (**C**) liver weight. Serum (**D**,**E**) and hepatic (**F**,**G**) level of triglycerides (TGs; **D**,**F**) and total cholesterol (TC; **E**,**G**). (**H**) relative mRNA expression of Hmgcr, (**I**) FXR protein expression with Vinculin as a loading control; relative mRNA expression of (**J**) Cyp27a1, (**K**) Abca1, (**L**) Srb1, (**M**) Npc1l1, and (**N**) Pgc1α, (**O**) Il1β, (**P**) Il6, and (**Q**) Tnfα were measured. Data are presented as means ± SD. When no significant interaction was detected by two-way ANOVA, US treatment effects were evaluated using Duncan’s post hoc test; different letters indicate significant differences among treatment groups within the same stress condition (*p* < 0.05). Differences between non-stress and UCMS within each treatment were assessed by Student’s *t*-test (^+^ *p* < 0.05, ^++^ *p* < 0.01; ^+++^ *p* < 0.001). W, water; US, sucralose; S, sucrose; UCMS, unpredictable chronic mild stress; NAFLD, non-alcoholic fatty liver disease; TG, triglyceride; TC, total cholesterol.

Chronic sucralose consumption maintained lower fasting blood glucose, insulin levels, and HOMA-IR scores compared to the S group (Figure S2). Histological analysis revealed that the US group exhibited significantly lower NAFLD activity scores than the S group, but significantly higher scores compared to the W group under both conditions (Figure 3A,B). These findings suggest that although sucralose produced less severe hepatic changes than sucrose, it was still associated with mild hepatic impairment.

Liver weight in the US group was comparable to W and lower than S under both conditions (Figure 3C). Serum TG and TC in US were not significantly elevated relative to W, and hepatic TG did not differ significantly between US and W (Figure 3D–F). In contrast, hepatic TC was significantly lower in US than in W, indicating that the clearest lipid difference relative to the water control was reduced hepatic cholesterol rather than a generalized reduction in hepatic lipids (Figure 3G). Note that the sucrose group showed significantly lower serum TG and TC and significantly higher hepatic TG and TC compared to US group, implying hepatic lipid accumulation as expected.

The regulatory loop between bile acid synthesis and its feedback control exhibited distinct steady-state patterns. Markers related to cholesterol synthesis, bile acid synthesis, bile acid signaling, and cholesterol trafficking exhibited distinct treatment- and stress-dependent patterns.

Hepatic *Hmgcr* mRNA expression, reflecting the transcriptional regulation of de novo cholesterol synthesis, in US was not significantly different from W under either non-stressed or chronic-stress conditions, although US was higher than S within each condition (Figure 3H).

In addition, the US group showed elevated FXR (farnesoid X receptor) protein, a bile acid-activated nuclear receptor that functions mainly as a sensor and regulator of the bile acid pool (Figure 3I) compared to the W group under non-stressed condition. However, UCMS exposure drastically downregulated FXR in the S and US groups, effectively silencing the feedback sensor, while the W group remained stable (Figure 3I). Despite this loss of FXR-mediated inhibition, the US group maintained high *Cyp27a1* levels, which contributes to the alternative bile acid synthesis pathway, compared to the S group, indicating dysregulation of bile acid homeostasis (Figure 3J).

For *Cyp27a1*, the S group showed significantly lower expression than both W and US under both non-stressed and chronic-stress conditions, whereas W and US did not differ significantly (Figure 3J).

Regarding cholesterol trafficking, hepatic *Abca1* and *Srb1* mRNA levels were higher in US than in both W and S under non-stressed and chronic-stress conditions (Figure 3K,L). A significant treatment-by-stress interaction was detected for *Srb1* (F = 6.511, *p* = 0.004), and *Srb1* increased further in the US group under chronic stress, indicating that stress-induced physiological shifts further enhance sucralose-associated changes in hepatic cholesterol handling (Figure 3L). Intestinal *Npc1l1* also showed a significant interaction (F = 3.763, *p* = 0.032). Under non-stressed conditions, *Npc1l1* was lower in US than in W, whereas under chronic stress, US was higher than W; importantly, *Npc1l1* did not change significantly between stress conditions within the US group. The significant within-treatment stress changes occurred in W and S, with a decrease in W and an increase in S (Figure 3M).

Furthermore, hepatic *Pgc1α* mRNA expression was significantly increased in the US group compared to both W and S groups (Figure 3N), potentially contributing to the observed reduction in hepatic lipid accumulation.

For inflammatory markers, *Il1β* in the US group was higher than in the S group but did not differ significantly from the W group under non-stressed conditions, and no treatment differences were evident under chronic stress (Figure 3O). *Il6* in the US group was higher than in both W and S groups under non-stressed conditions, whereas under chronic stress, S was higher than US and W was intermediate (Figure 3P). Tnfα in in the US group was higher than in both W and S groups under non-stressed conditions; under chronic stress, S was higher than W and US was intermediate (Figure 3Q). Significant treatment-by-stress interactions for *Il6* (F = 12.282, *p* < 0.001) and Tnfα (F = 6.854, *p* = 0.003) indicate that the inflammatory response depends on stress condition. Overall, the data show reduced hepatic cholesterol together with altered cholesterol trafficking and stress-sensitive inflammatory regulation, rather than a uniform reduction in hepatic lipid accumulation.

### 3.4. Long-Term Sucralose Consumption Altered the Colon Barrier and Induced Inflammation with XBP1-Dependent ER Stress

**Figure 4:**
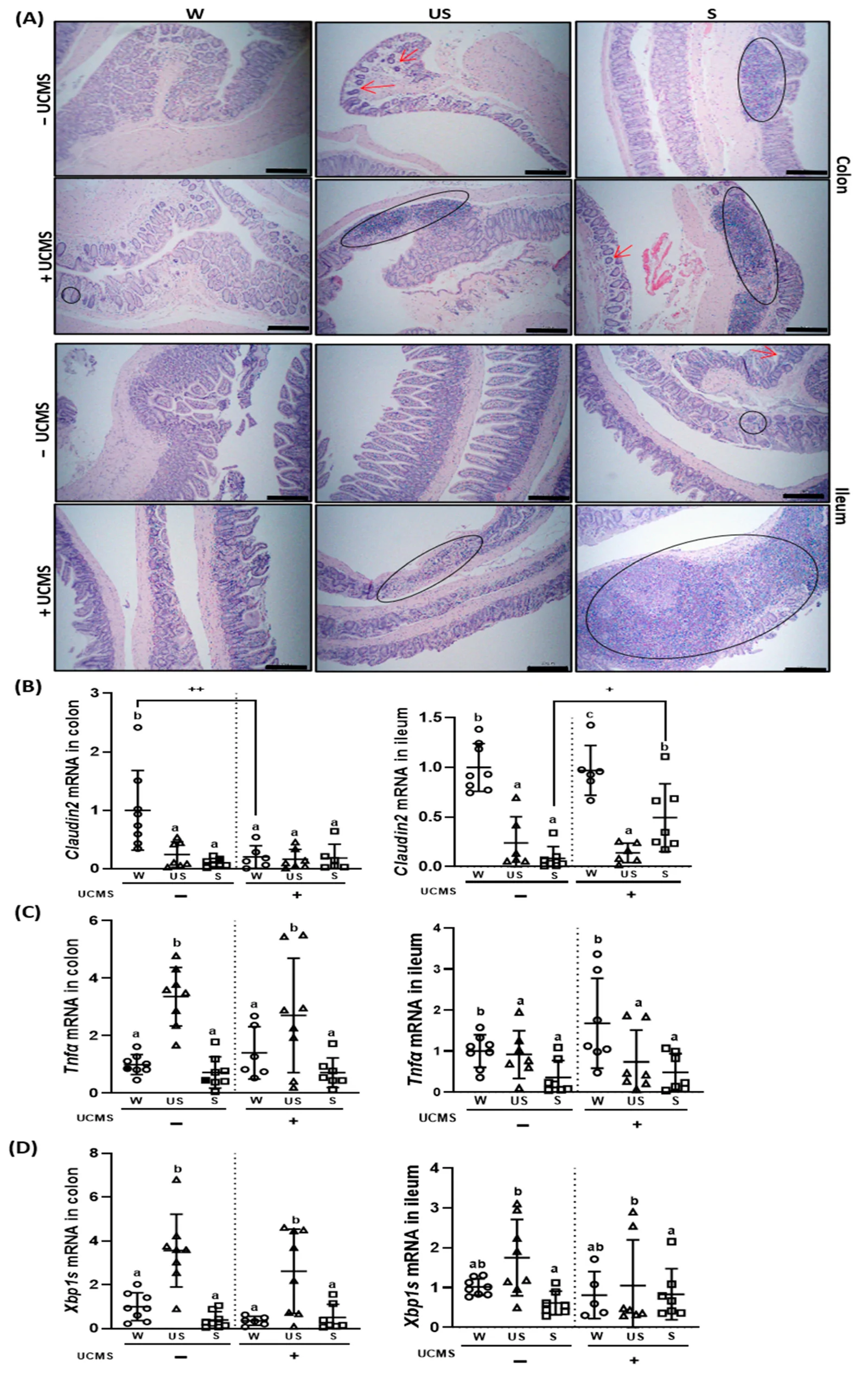
The impact of sucralose and chronic stress on intestinal barrier integrity, inflammation, and ER stress. (**A**) Representative H&E-stained sections (10× magnification; scale bar: 200 µm). Black circles indicate inflammatory cell infiltration; red arrows indicate the loss of crypts and the epithelial damage. Relative mRNA expression of (**B**) Claudin2, (**C**) Tnfα, and (**D**) Xbp1s in colon and ileum tissues were measured by *qRT-PCR*. Data are presented as means ± SD. When no significant interaction was detected by two-way ANOVA, US treatment effects were evaluated using Duncan’s post hoc test; different letters indicate significant differences among treatment groups within the same stress condition (*p* < 0.05). Differences between non-stress and UCMS within each treatment were assessed by Student’s *t*-test (^+^ *p* < 0.05, ^++^ *p* < 0.01). W, water; US, sucralose; S, sucrose; UCMS, unpredictable chronic mild stress.

Human pharmacokinetic data indicate that approximately 15% of an oral sucralose dose is systemically absorbed, with most of the dose recovered in feces [44]. We therefore examined both the colon and ileum because of their distinct roles in mucosal defense and immune surveillance [45]. Histological examination under non-stress conditions revealed that comparable levels of crypt loss and epithelial damage in the US and S groups (Figure 4A). However, under chronic stress, the US group exhibited more pronounced inflammatory cell infiltration, suggesting that long-term sucralose consumption might amplify structural gut alterations when combined with stress.

Long-term sucralose consumption consistently disrupted intestinal barrier markers, with effects modulated by region and stress. In the colon, *Claudin2* expression was significantly reduced in the US and S groups compared to the W group under non-stressed conditions (Figure 4B). A significant interaction between treatment and stress was observed (*F* = 6.133, *p* = 0.005), driven by a sharp decline in these markers within the W group under stress. This suggests that chronic stress itself compromises colonic barrier integrity to a degree comparable to sweetener consumption. In the ileum, *Claudin2* expression also showed a significant interaction (*F* = 4.610, *p* = 0.017); while unexpectedly increasing its expression in the S group, the US group showed persistent downregulation regardless of stress, indicating a chronic, stress-independent negative effect of sucralose on ileal tight junctions.

*Tnfα* expression was consistently elevated in the colon of the US group relative to both controls, whereas in the ileum, UCMS significantly upregulated *Tnfα* expression in the W group (Figure 4C). We examined expression of the spliced Xbp1 form, *Xbp1s*, a marker of ER stress-induced IRE1 pathway activation. *Xbp1s* expression of the US group was significantly upregulated in the colon compared to both W and S groups (Figure 4D). In the ileum, however, XBP1s was significantly upregulated compared to only the S group although there was no difference between the US group and W group under both non-stressed and stressed condition. Interestingly, this response was tissue-specific, as no significant changes in *Xbp1s* mRNA were detected in the liver, suggesting that the gut is a primary site for sucralose-induced ER stress. These findings indicate that long-term sucralose consumption may disrupt gut barrier integrity and trigger region-specific inflammatory and ER stress responses, which are selectively exacerbated or masked by chronic stress conditions.

### 3.5. Long-Term Sucralose Consumption Altered the Gut Microbiota

**Figure 5:**
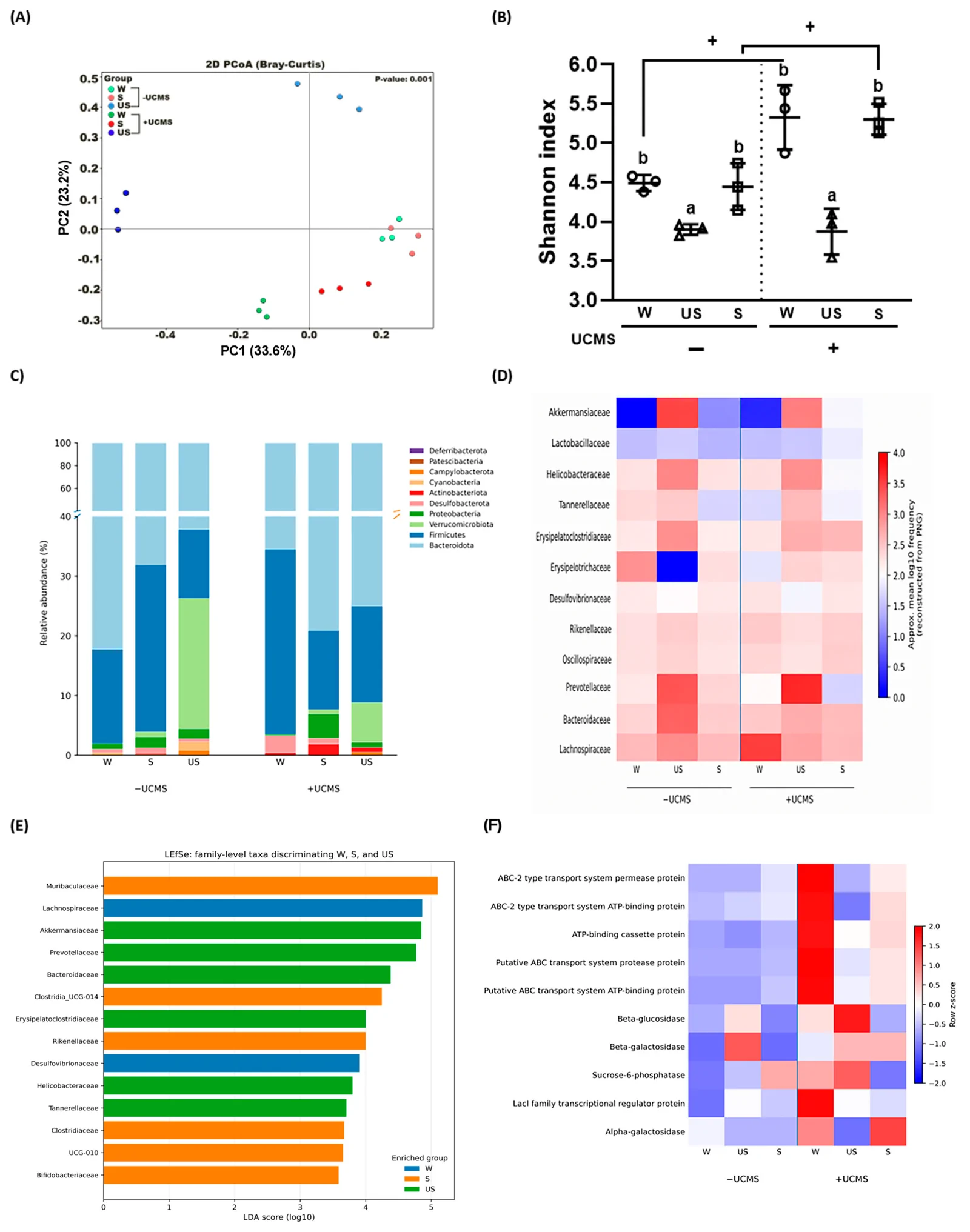
Alterations in gut microbiome diversity, taxonomic composition, and predicted microbial function. (**A**) Beta diversity of fecal microbial communities based on Bray–Curtis dissimilarity. (**B**) Alpha diversity assessed using the Shannon index. (**C**) Relative taxonomic composition at the phylum level. (**D**) Family-level taxonomic profile. (**E**) LEfSe-derived linear discriminant analysis (LDA) effect-size scores for family-level taxa discriminating the W, S, and US groups across non-stressed and UCMS conditions. Treatment was specified as the main class and stress condition as the subclass; taxa with Kruskal–Wallis *p* < 0.05 and LDA score > 2.0 are shown. (**F**) Predicted selected KEGG functional pathways. Data are presented as the means ± SD. When no significant interaction was detected by two-way ANOVA, US treatment effects were evaluated using Duncan’s post hoc test; different letters indicate significant differences among treatment groups within the same stress condition (*p* < 0.05). Differences between non-stress and UCMS within each treatment were assessed by Student’s *t*-test (^+^ *p* < 0.05). W, water; US, sucralose; S, sucrose; UCMS, unpredictable chronic mild stress.

To explore whether the observed intestinal barrier changes were accompanied by changes in the gut microbiota, we analyzed fecal microbial composition and predicted functional profiles. Beta diversity analysis based on Bray–Curtis dissimilarity revealed a significant separation of microbial communities according to treatment and stress conditions (PERMANOVA, *p* = 0.001; Figure 5A). The US group exhibited the most pronounced separation from other groups, while the W and S groups remained relatively clustered under baseline conditions. Stress exposure triggered clear shifts in all groups, with the US group showing the most substantial longitudinal separation, indicating a unique and sensitive microbial signature.

Sucralose consumption significantly reduced alpha diversity (Shannon index) under both conditions (Figure 5B). While chronic stress increased alpha diversity in the W and S groups (*F* = 5.797, *p* = 0.017), the US group did not exhibit this response, maintaining consistently low diversity.

Representative taxonomic profiling at the phylum level further demonstrated distinct microbial patterns among the groups (Figure 5C). Under non-stressed conditions, the US group showed a pronounced enrichment of Verrucomicrobiota and Cyanobacteria accompanied by a lower relative contribution of Firmicutes compared with the W and S groups. Following UCMS exposure, Verrucomicrobiota remained relatively enriched in the US group, whereas the microbial composition of the S group showed a marked increase in Proteobacteria. These findings indicate that sucralose consumption produced a distinct phylum-level microbial profile that persists, at least in part, during chronic stress.

To further characterize microbial taxa potentially relevant to the gut–liver axis, selected bacterial families associated with intestinal barrier function, microbial metabolism and host metabolic regulation were examined (Figure 5D, Full family taxa in Figure S3A). The US group showed a unique enrichment of Akkermansiaceae, Helicobacteraceae, Erysipelatoclostridiaceae, Bacteroidaceae and Prevotellaceae under non-stressed conditions. UCMS markedly altered several additional family-level patterns, including Akkermansiaceae, Helicobacteraceae, Prevotellaceae, and Tannerellaceae. These findings demonstrate that both sucralose consumption and chronic stress are associated with restructuring of bacterial families potentially relevant to gut–liver metabolic communication.

LEfSe analysis was additionally performed at the family level to identify bacterial taxa that discriminated the three treatment groups while maintaining a consistent direction across non-stressed and UCMS conditions (Figure 5E). Fourteen bacterial families exceeded the prespecified LDA threshold of 2.0. The strongest discriminator for the S group was Muribaculaceae (LDA = 5.10), followed by Clostridia_UCG-014 (LDA = 4.25), Rikenellaceae (LDA = 4.00), Clostridiaceae (LDA = 3.67), UCG-010 (LDA = 3.65), and Bifidobacteriaceae (LDA = 3.59). The W group was characterized primarily by Lachnospiraceae (LDA = 4.86) and Desulfovibrionaceae (LDA = 3.90). In contrast, the US group showed a distinct set of discriminative families, including Akkermansiaceae (LDA = 4.85), Prevotellaceae (LDA = 4.77), Bacteroidaceae (LDA = 4.38), Erysipelatoclostridiaceae (LDA = 4.00), Helicobacteraceae (LDA = 3.80), and Tannerellaceae (LDA = 3.71). These treatment-associated signatures were directionally consistent across the two stress strata, indicating that long-term sucralose exposure was associated with a reproducible family-level restructuring of the fecal microbiota rather than with a pattern restricted to a single stress condition.

Functional predictions using KEGG pathway analysis further distinguished treatment- and stress-dependent differences in microbial metabolic potential. Analysis of selected biologically relevant KEGG orthologs showed prominent alterations in ATP-binding cassette (ABC) transport-related functions and carbohydrate-processing enzymes (Figure 5F, full KEGG pathway in Figure S3B). Under non-stressed conditions, the US group exhibited a functional profile distinct from both the W and S groups, including differences in carbohydrate-processing enzymes such as β-galactosidase and β-glucosidase. UCMS induced substantial functional reorganization, particularly between the W and US groups, with marked change in ABC transport functions. These findings indicate that long-term sucralose intake, unlike sugar, drives a specific functional shift in alternative carbohydrate processing, potentially contributing to the observed systemic metabolic dysregulation.

### 3.6. Long-Term Sucralose Consumption Increases Depressive-like Behaviors with Neuroinflammation

**Figure 6:**
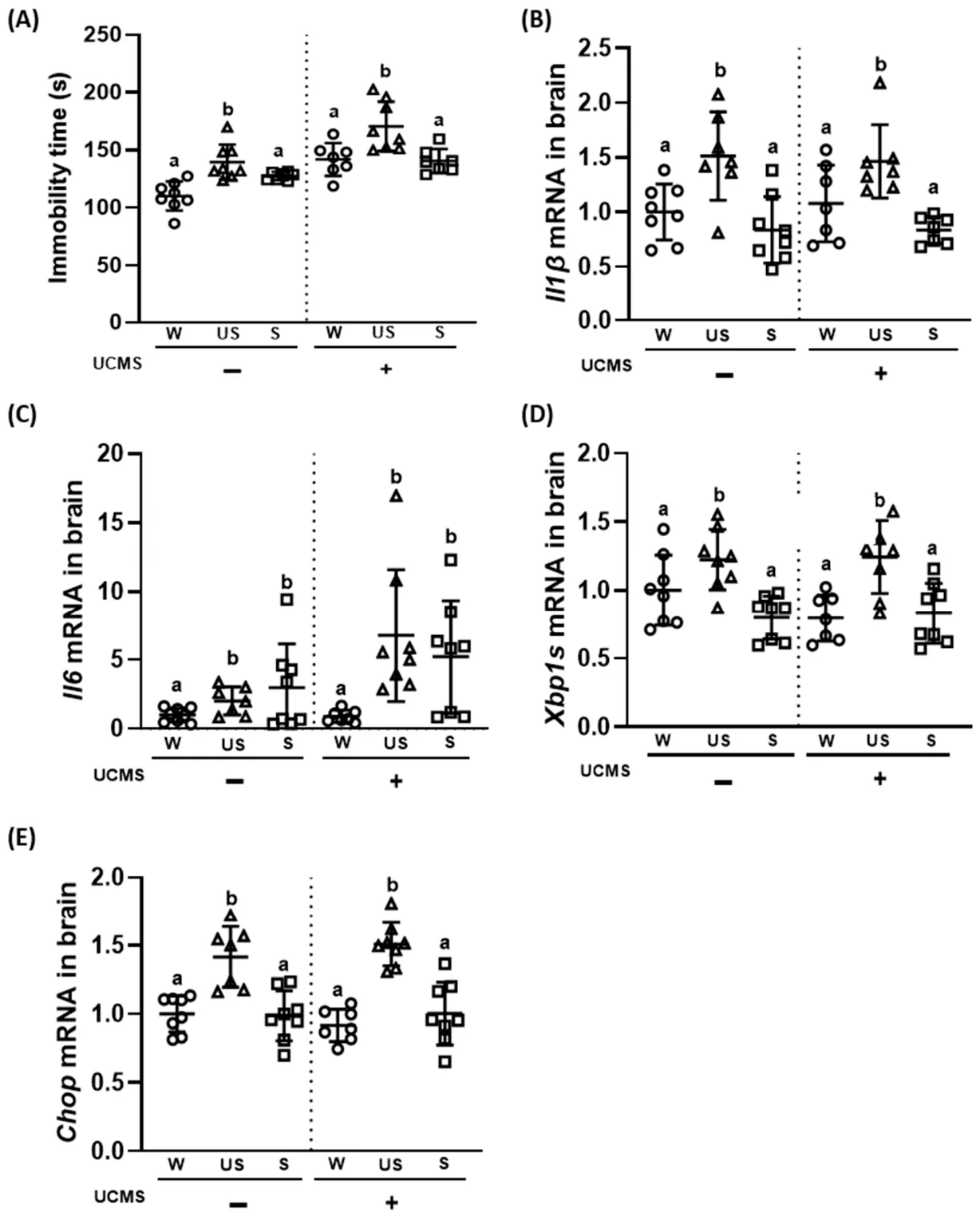
Sucralose increases depressive-like behaviors and neuroinflammation in mice. (**A**) Immobility time in the FST. Relative mRNA expression of (**B**) *Il1β*, (**C**) *Il6*, (**D**) *Xbp1s*, and (**E**) *Chop* were normalized to β-actin. Data are presented as means ± SD. When no significant interaction was detected by two-way ANOVA, US treatment effects were evaluated using Duncan’s post hoc test; different letters indicate significant differences among treatment groups within the same stress condition (*p* < 0.05). W, water; S, sucrose; US, sucralose; UCMS, unpredictable chronic mild stress.

To explore how sucralose-induced peripheral shifts influence neurological outcomes, we conducted behavioral assessments. In the Forced Swim Test (FST), the US group exhibited significantly increased immobility time compared to both the W and S groups under both conditions, signaling the induction of a robust depressive-like phenotype (Figure 6A).

Therefore, proinflammatory gene expression and ER stress markers in the brain were analyzed to identify potential neurobiological drivers of these behaviors. *Il1β* expression was significantly elevated in the US group compared with the S group under both conditions (Figure 6B). Similarly, *Il6* levels were consistently greater in the US group than in the W group (Figure 6C). Furthermore, analysis of ER stress pathways revealed a significant upregulation of *Xbp1s* and *Chop* in the US group relative to the S group across both conditions (Figure 6D,E). These results indicate that neuroinflammation and activation of XBP1s-CHOP-associated ER stress signaling may contribute to neuronal dysfunction, potentially contributing to the observed depressive-like behaviors.

### 3.7. Correlation Analysis of Sucralose-Induced Effects and Neurobehavioral Outcomes

**Figure 7:**
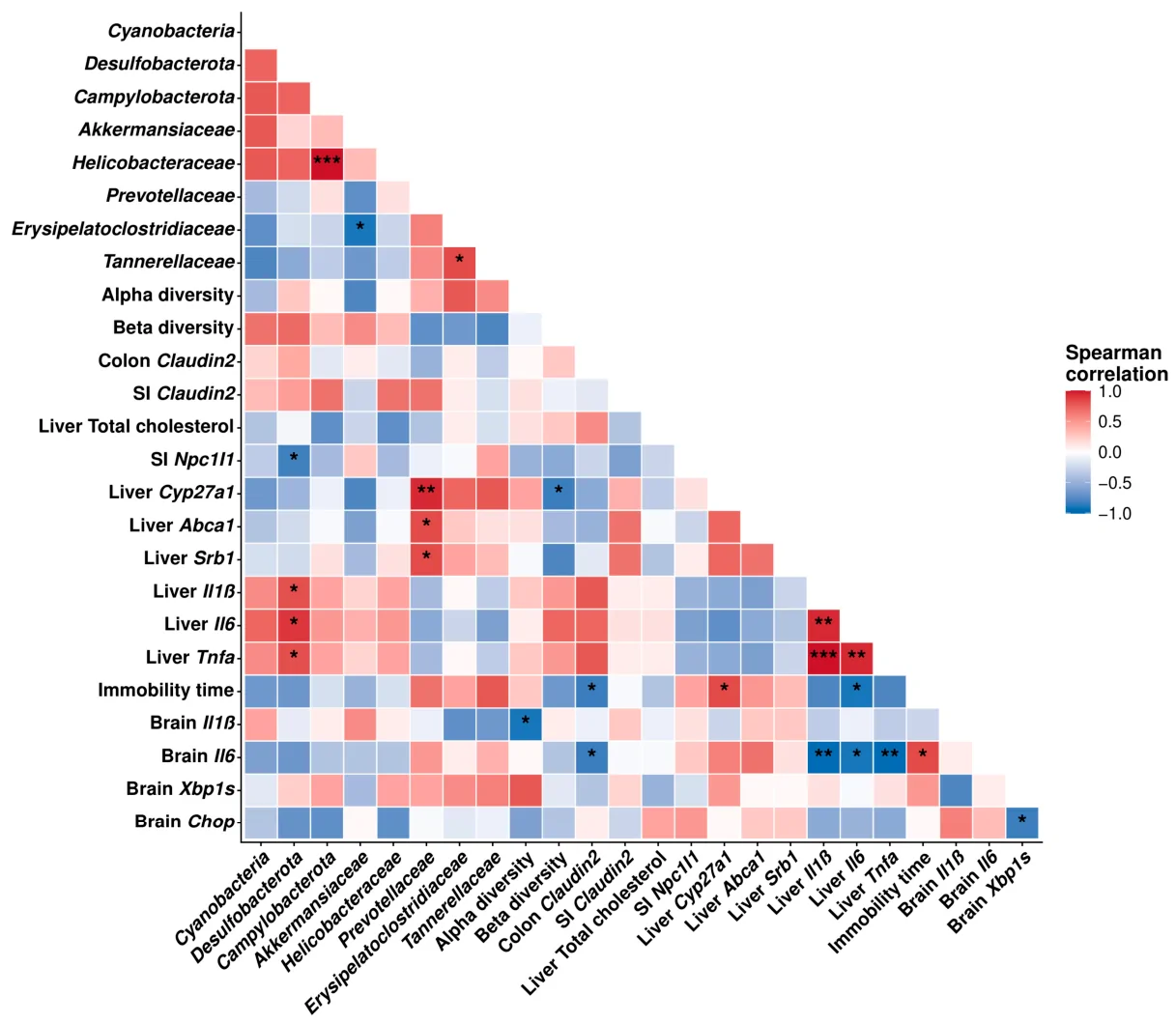
Integrated Spearman correlation analysis of the gut–liver–brain axis. A correlation heatmap illustrating the integrative analysis of Spearman’s rank correlation coefficients (ρ) within the sucralose-treated groups. The matrix displays association among microbial taxa, intestinal barrier integrity markers, hepatic metabolic and inflammatory indicators, and neurobehavioral outcomes. The color scale represents the strength of the correlation, with red and blue tiles indicating positive and negative correlation, respectively. Asterisks denote statistical significance (* *p* < 0.05; ** *p* < 0.01; *** *p* < 0.001).

To investigate the complex association among the sucralose-induced physiological shifts, we performed Spearman’s rank correlation analysis specifically within the sucralose-treated (US) groups (Figure 7). Among microbial taxa, Prevotellaceae showed significant positive correlations with markers of hepatic cholesterol metabolism including *Cyp27a1*, *Abca1*, and *Srb1*. Desulfobacterota abundance was positively correlated with liver inflammatory markers (*Il1β*, *Il6* and *Tnfα*), while showing a negative correlation with the intestinal *Npc1l1* gene.

In terms of intestinal integrity, colonic *Claudin2* expression was negatively associated with immobility time and brain *Il6*. In addition, alpha diversity was negatively associated with brain *Il1β*, suggesting a potential link between microbial community stability and the mitigation of neuroinflammation.

Hepatic markers also showed a strong association with central nervous system responses. Notably, the hepatic *Cyp27a1* gene was positively correlated with immobility time, reflecting a potential gut–liver–brain link. Overall, these findings provide a comprehensive map of the gut–liver–brain axis, highlighting how sucralose-induced dysbiosis and peripheral organ dysfunction are integrated with altered neurobehavioral states.

## 4. Discussion

Consumption of reduced- and no-sugar beverages has been documented in several regions. In South Korea, per capita consumption of zero- or low-calorie carbonated beverages increased from 4.1 g/day in 2019 to 21.9 g/day in 2023 [46]. In the United States, diet beverages accounted for 2.0% of total non-alcoholic beverage intake among youth and 4.8% among adults in NHANES 2021–2023 [47]. A review of national dietary surveys in the WHO European Region estimated mean intakes of reduced- or no-sugar soft drinks at approximately 44 g/day in children and 36 g/day in adults [48], although estimates varied across countries and survey definitions. These population data indicate consumption of reduced- and no-sugar beverages, but do not establish the intake of any specific sweetener, including sucralose, or the proportion of consumers approaching its ADI. Accordingly, the dose used in the present study was selected as an ADI-level experimental benchmark, rather than as an estimate of typical human exposure.

The main objective of this study was to investigate how long-term sucralose consumption from early-life affects metabolic health and neurobehavioral outcomes. Our findings demonstrated coordinated alterations across the gut–liver–brain axis. Consistent with previous studies, sucralose did not increase body weight [12,13], although this contrasts with evidence associating long-term sucralose consumption with obesity, adiposity, and insulin resistance [8].

Despite concerns that sucralose may promote obesity [9], total fat percentage and adipose depot weights were lower in sucralose-treated mice than in sugar controls, consistent with studies reporting minimal effects on body weight or adiposity [12,49], but contrasting with increased subcutaneous abdominal fat reported in sucralose-treated mini-pigs [50]. eWAT histology confirmed no marked adipocyte hypertrophy. Conversely, sucralose has been reported to promote adipogenesis and increase *Pparγ* and *Fas* expression in human adipocytes and 3T3-L1 cells [10,51]. In our study, however, *Pparγ* was downregulated while *Fas* was increased, suggesting enhanced lipogenic signaling without increased adipocyte differentiation. Sucralose also induced depot-specific inflammation, with increased *Il6* in eWAT and *Tnfa* in visceral adipose tissues, consistent with reported dysregulation of *Tnfa* -related signaling in human adipose tissue [52]. Thus, chronic sucralose consumption may alter lipid metabolism and inflammatory signaling despite reduced adiposity.

Serum triglyceride and total cholesterol were not significantly increased by sucralose relative to the water control, and hepatic triglyceride was also unchanged. In contrast, hepatic cholesterol was lower in the US group under both non-stressed and chronic-stress conditions, and *Hmgcr* expression was higher in the US group than in the S group but was not significantly different from the W group; therefore, these data do not demonstrate a clear compensatory increase in de novo cholesterol synthesis relative to the water control. Hepatic *Abca1* and *Srb1*, involved in cholesterol efflux and HDL-derived cholesterol flux, respectively, were increased in the US group relative to both controls, whereas intestinal *Npc1l1* showed a treatment-by-stress interaction. Together, these findings indicate altered cholesterol trafficking and distribution rather than generalized reduction in hepatic lipid accumulation.

Changes in hepatic cholesterol were accompanied by altered bile acid-related markers. FXR functions as a bile acid sensor and regulator of bile acid homeostasis [53]. *Cyp27a1* expression was lower in the S group than in the W and US groups under both conditions, whereas the W and US groups did not differ significantly. Because cholesterol is also as a precursor for other essential molecules such as vitamin D and steroid hormones [54,55], which were not assessed in this study, the functional consequences of reduced hepatic cholesterol remain uncertain. In addition, chronic hepatic inflammation can contribute to metabolic dysfunction and fatty liver disease [56], indicating that lower hepatic cholesterol should not automatically be interpreted as metabolically beneficial.

Increased brain *Il1β* expression under both non-stressed and stressed conditions further supports an association between chronic sucralose intake and neuroinflammatory signaling [57]. Although sucralose independently affected hepatic and cerebral inflammatory markers, correlation analysis did not reveal a prominent direct relationship between hepatic *Il1β* and brain *Il6*, suggesting that neuroinflammation may involve tissue-specific rather than simple linear inflammatory pathways.

Reduced alpha diversity in our study supports studies showing microbial disruption following sucralose exposure [58,59,60]. Our finding of reduced microbial diversity is also consistent with studies associating lower diversity with metabolic and intestinal dysfunction [61,62].

LEfSe analysis further clarified the treatment-specific microbial characteristics observed in the analyses of diversity and taxonomic-genomic composition. The water control group was characterized by the Lachnospiraceae and Desulfovibrionaceae families, whereas the sucrose group was most notably distinguished by Muribaculaceae and several additional Clostridia- and carbohydrate-related families. In contrast, long-term sucralose exposure resulted in a broader range of distinguishable taxa, including Akkermansiaceae, Prevotellaceae, Bacteroidaceae, Erysipelatoclostridiaceae, Helicobacteraceae, and Tannerellaceae. Tannerellaceae and Erysipelatoclostridiaceae, were enriched in the sucralose group. Tannerellaceae has been reported as differentially abundant in an experimental autoimmune hepatitis model, whereas Erysipelatoclostridiaceae has been reported as enriched in a Korean colorectal cancer cohort [63,64]. Notably, these taxa maintained the same direction of enrichment in both the non-stressed and UCMS groups, suggesting that the microbial characteristics associated with sucralose were not determined solely by stress conditions.

The composition of these signatures indicates a restructuring of various ecological niches involved in host–microbe interactions. Specifically, the concurrent differentiation of Akkermansiaceae, Prevotellaceae, and Bacteroidaceae aligns with altered utilization of mucin- and carbohydrate-derived substrates; meanwhile, the proliferation of Erysipelatoclostridiaceae, Helicobacteraceae, and Tannerellaceae may reflect broader changes in the internal environment associated with the barrier and inflammatory alterations observed in this study. Conversely, the relative predominance of Lachnospiraceae in the water control group suggests that sucralose exposure was accompanied by shifts in microbial community structure. This phenomenon contrasts with the characteristic microbial structure observed in untreated animals. Thus, these results extend the heatmap analysis by identifying the specific bacterial lineages that contribute most significantly to the treatment-associated differentiation.

Nevertheless, the LEfSe results should be interpreted as treatment-associated microbial signatures rather than direct evidence of beneficial or pathogenic functions. The analysis was conducted at the family level, and significant functional heterogeneity can exist between genera and species within the same family. Furthermore, 16S rRNA-based taxonomic profiling cannot elucidate microbial metabolic activity or establish causal links with hepatic, cardiac, or behavioral phenotypes. Therefore, while these findings support a strong association between long-term sucralose consumption and the restructuring of the gut microbiota, further research, such as shotgun metagenomic analysis, targeted metabolomic profiling, or microbial transplantation experiments, is required to determine the functional implications of these taxonomic changes.

Consistent with the hypothesis that adverse changes in the gut environment may exacerbate neuroinflammation and behavioral changes through the gut–brain axis, our study revealed that sucralose exposure induced marked neuroinflammatory responses and behavioral changes. The results of the behavioral analyses in this study revealed that long-term sucralose consumption from the early-life stage was associated with prominent depressive-like phenotypes under both non-stressed and UCMS conditions. The significant increase in immobility time during the forced swim test aligns with evidence indicating that gut function and inflammatory disruptions can drive depressive-like behaviors [65,66].

In addition, the functional pathway predictions in our study revealed an increased abundance of carbohydrate-active enzymes, including β-galactosidase and β-glucosidase, which may increase luminal carbohydrate metabolism [67,68]. Taken together, our results provide evidence that long-term sucralose consumption compromises the intestinal barrier and changes the composition and metabolic capacity of the gut microbiome, changes that may contribute to long-term gut and systemic health risks.

Although our findings strongly support an association between long-term sucralose consumption, gut microbial dysbiosis, intestinal barrier disruption, systemic inflammation, and neurobehavioral alterations, this study does not directly link the gut to the brain. Therefore, future functional studies such as fecal microbiota transplantation from sucralose-treated mice into germ-free or antibiotic-treated recipient mice are needed to confirm the observed metabolic, inflammatory, and behavioral phenotypes within the gut–liver–brain axis.

Although chronic stress is known to activate the hypothalamic–pituitary–adrenal (HPA) axis and it was not directly assessed in the present study, the observed stress-dependent exacerbation of gut barrier disruption, inflammatory signaling, and behavioral outcomes is consistent with known HPA axis-mediated modulation of metabolic and neuroimmune pathways, and it would be worthwhile to investigate further in the future [69,70,71,72].

Future studies should examine sex differences, dose–response relationships spanning regulatory exposure ranges in different regions, HDL and LDL fractions, metabolomic profiles including short-chain fatty acids and bile acids [73], and targeted interventions aimed at restoring gut barrier integrity. Studies that model common commercial formulations, including combinations with other sweeteners or bulking agents, would also improve relevance to real-world dietary exposure. Translation to humans will require controlled clinical intervention studies, which to date have reported heterogeneous metabolic and microbiome responses to sucralose [74,75,76,77], as well as additional toxicological work, particularly because the present study used a sustained mouse exposure paradigm rather than a direct model of habitual human intake.

Our study reveals potential for diet–stress interactions to exacerbate vulnerability to mood disorders and cognitive dysfunction. To our knowledge, this study is the first to investigate the comprehensive impact of long-term consumption of sucralose and chronic stress from the early-life stage on metabolic and behavioral markers, emphasizing the novelty of this approach and its contribution to a growing and fragmented field.

Based on our findings, long-term sucralose consumption was associated with altered gut microbial composition and barrier markers, hepatic cholesterol handling, inflammatory signaling, and neurobehavioral outcomes. These changes occurred across gut-, liver-, brain-related pathways, but the present design does not establish causal communication across a gut–liver–brain axis. The U.S. FDA and EFSA currently consider sucralose acceptable within their respective ADIs, which differ in magnitude [5,6]. Accordingly, our preclinical findings should be viewed as a rationale for additional mechanistic, toxicological, and controlled clinical intervention studies rather than as evidence that currently authorized human use is unsafe.

## 5. Conclusions

Long-term sucralose consumption did not promote weight gain or overt glucose dysregulation, but it was associated with changes in hepatic cholesterol handling, inflammatory signaling, intestinal barrier markers, gut microbial composition, neuroinflammatory markers, ER stress, and depressive-like behavior. The most consistent lipid finding relative to the water control was reduced hepatic cholesterol, accompanied by altered *Abca1* and *Srb1* expression and a stress-dependent pattern of intestinal *Npc1l1*, suggesting that the consequences of chronic sucralose exposure extend beyond local hepatic metabolism. The integrated findings indicate that long-term sucralose intake may induce metabolic reprogramming despite the absence of obesity or insulin resistance.

## Data Availability

The original contributions presented in the study are included in the article/Supplementary Material, and further inquiries can be directed to the corresponding author.

## Supplementary Materials

The following supporting information can be downloaded at: https://www.mdpi.com/article/10.3390/nu18203452/s1, Table S1: Experimental design and UCMS protocol; Figure S1: Long-term consumption of sucralose did not increase adipose tissue mass; Figure S2: Long-term consumption of sucralose did not increase blood glucose or insulin levels; Figure S3: Alterations in gut microbiome profile.

## Abbreviations

The following abbreviations are used in this manuscript:

*Il1β*	Interleukin-1 beta
*Tnfα*	Tumor Necrosis Factor alpha
HMG-CoA reductase	3-hydroxy-3-methylglutaryl-coenzyme A reductase
*Cyp27a1*	Cytochrome P450 family 27 subfamily A member 1
FXR	Farnesoid X Receptor
*Abca1*	ATP Binding Cassette Subfamily A Member 1
HDL	High-density lipoprotein
LDL	Low-density lipoprotein
*Muc1*	Mucin 1
*Xbp1s*	Spliced X-box binding protein 1
*Chop*	C/EBP homologous protein
HAD super family	Haloacid dehydrogenase (HAD) superfamily
KEGG pathway	Kyoto Encyclopedia of Genes and Genomes pathway
ER stress	Endoplasmic Reticulum stress
mRNA	messenger ribonucleic acid
SCFA	Short-chain fatty acid
