# Supplementary material for: Long-Term Sucralose Consumption Alters Gut Microbiota and Hepatic Lipid Metabolism and Increases Depressive-like Behavior in Young Mice
Source: Nutrients. 2026 Oct 21;18(20):3452. doi: 10.3390/nu18203452 (PMC13708054; doi:10.3390/nu18203452)
Supplement: Supplementary file 1 [file nutrients-18-03452-s001.zip › nutrients-4553377-supplementary.pdf]

Supplementary Material

Table S1. Experimental design and UCMS protocol

	4	5	6	7	8	9	10	11	12	13	14	15	16	17	18
Monday		LDR	SC	LDR	CWS	WWS	CWS	EC	R	TP	WWS	SC	FST	WC	MWM
Tuesday		LDR	CWS	LDR	TP	SC	LDR	TP	SC	R	TP	FD	FST	LDR	MWM
Wednesday		R	CT	WWS	R	TP	LDR	SC	WWS	CWS	R	OFT	FD	LDR	Sacrifice
Thursday	WC	TP	FD	WC	LDR	EC	CT	FD	LDR	WC	LDR	OFT	SC	R	
Friday	CWS	WWS	R	SC	LDR	CWS	R	WWS	LDR	SC	LDR	R	EC	MWM	
Saturday	EC	EWB	WC	CT	EC	EWB	FD	CT	WC	CT	EC	EWB	CT	MWM	
Sunday	FD	CT	EC	EWB	FD	CT	WC	EWB	EC	FD	SC	CT	WWS	MWM	

FD, food deprivation; WC, wet cage; CWS, cold water swimming; EC, empty cage; CT, cage tilt; LDR, light and dark cycle reversal; R, restriction; TP, tail pinch; WWS, warm water swimming; EWB, empty water bottle; SC, shaking cage administration; OFT, open field test; FST, forced swim test; MWM, Morris water maze

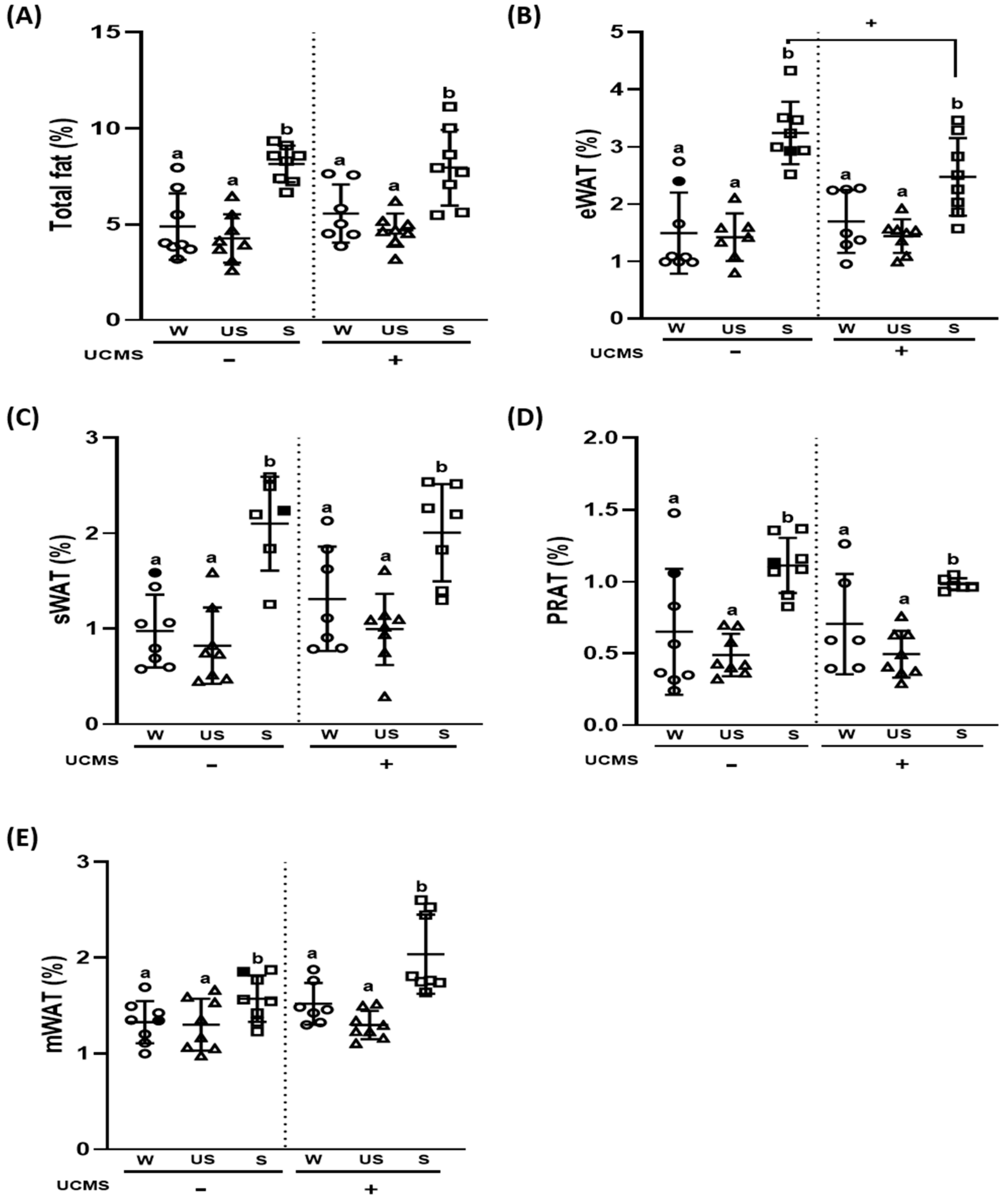

Figure S1. Long-term consumption of sucralose did not increase adipose tissue mass

Weight percentages of (A) total fat, (B) eWAT, (C) sWAT, (D) PRAT, (E) mWAT relative to total body weight were calculated. Data are presented as the means ± SD. When no significant interaction was detected by two-way ANOVA, US treatment effects were evaluated using Duncan's post-hoc test; different letters (a-c) indicate significant differences among treatment groups within the same stress condition ($p < 0.05$). Differences between non-stress and UCMS within each treatment were assessed by Student's t-test ($^{+}p < 0.05$). eWAT, epididymal white adipose tissue; sWAT, subcutaneous white adipose tissue; mWAT, mesenteric white adipose tissue; PRAT, perirenal adipose tissue; W, water; US, sucralose; S, sucrose; UCMS, unpredictable chronic mild stress.

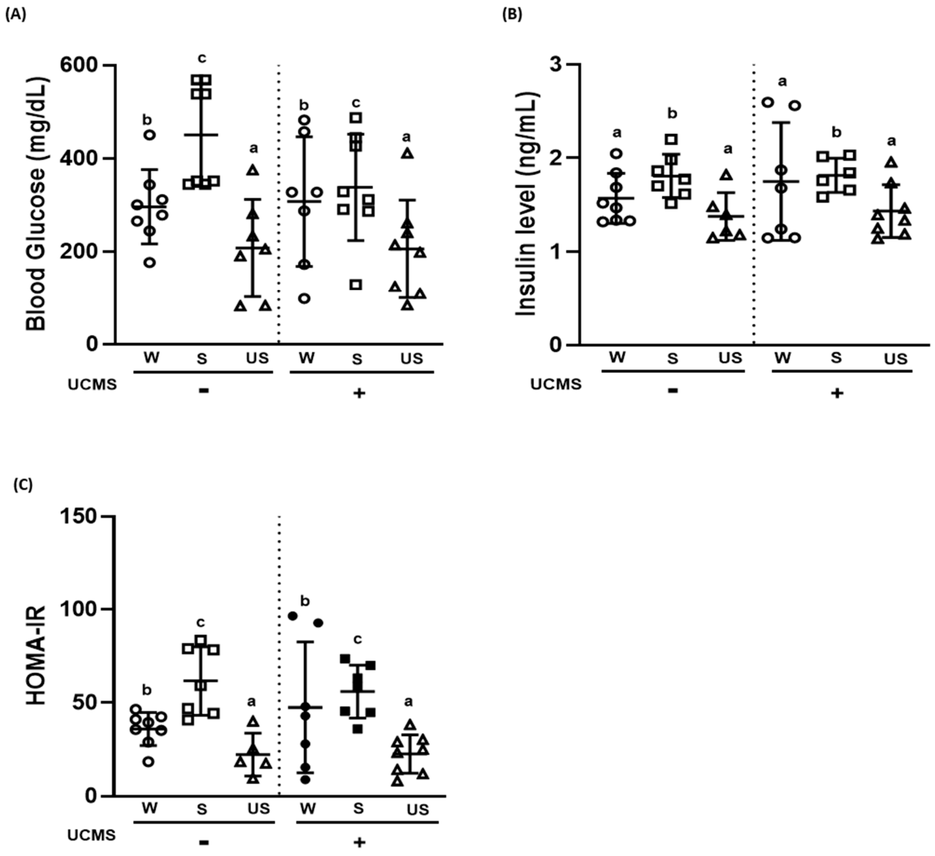

Figure S2. Long-term consumption of sucralose did not increase blood glucose or insulin levels

Serum levels of (A) glucose and (B) insulin were assessed using ELISA. (C) HOMA-IR insulin resistance was calculated as an index of insulin resistance. Data are presented as the means ± SD. When no significant interaction was detected by two-way ANOVA, US treatment effects were evaluated using Duncan's post-hoc test; different letters (a-c) indicate significant differences among treatment groups within the same stress condition ($p < 0.05$). W, water; US, sucralose; S, sucrose; UCMS, unpredictable chronic mild stress.

(A)

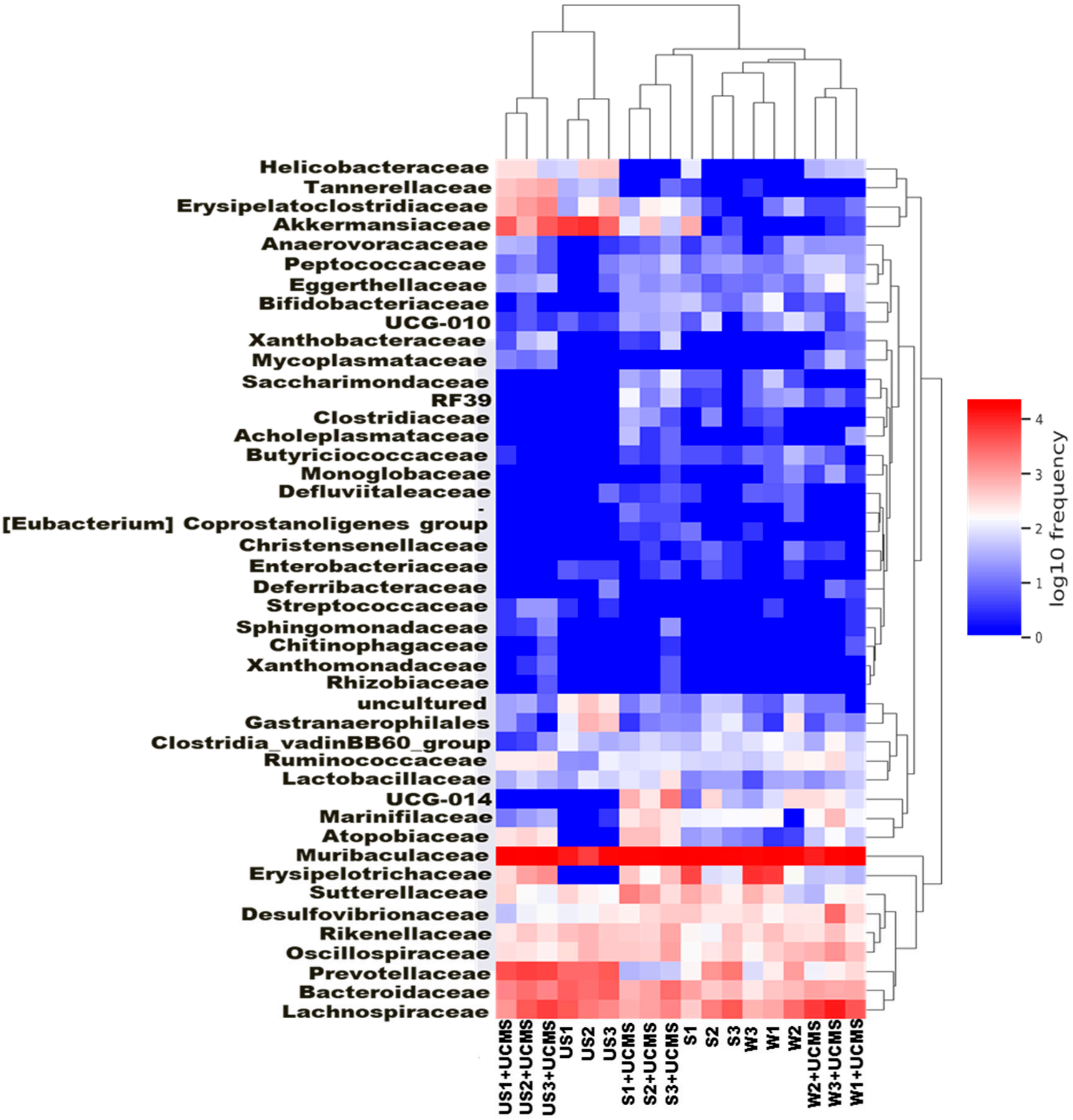
Helicobacteraceae
Tannerellaceae
Erysipelatoclostridiaceae
Akkermansiaceae
Anaerovoracaceae
Peptococcaceae
Eggerthellaceae
Bifidobacteriaceae
UCG-010
Xanthobacteraceae
Mycoplasmataceae
Saccharimondaceae
RF39
Clostridiaceae
Acholeplasmataceae
Butyriciococcaceae
Monoglobaceae
Defluviitaleaceae
-
[Eubacterium] Coprostanoligenes group
Christensenellaceae
Enterobacteriaceae
Deferribacteraceae
Streptococcaceae
Sphingomonadaceae
Chitinophagaceae
Xanthomonadaceae
Rhizobiaceae
uncultured
Gastranaerophilales
Clostridia_vadinBB60_group
Ruminococcaceae
Lactobacillaceae
UCG-014
Marinifilaceae
Atopobiaceae
Muribaculaceae
Erysipelotrichaceae
Sutterellaceae
Desulfovibrionaceae
Rikenellaceae
Oscillospiraceae
Prevotellaceae
Bacteroidaceae
Lachnospiraceae
US1+UCMS
US2+UCMS
US3+UCMS
US1
US2
US3
S1+UCMS
S2+UCMS
S3+UCMS
S1
S2
S3
W3
W1
W2
W2+UCMS
W3+UCMS
W1+UCMS
4
3
2
1
0
log10 frequency

(B)

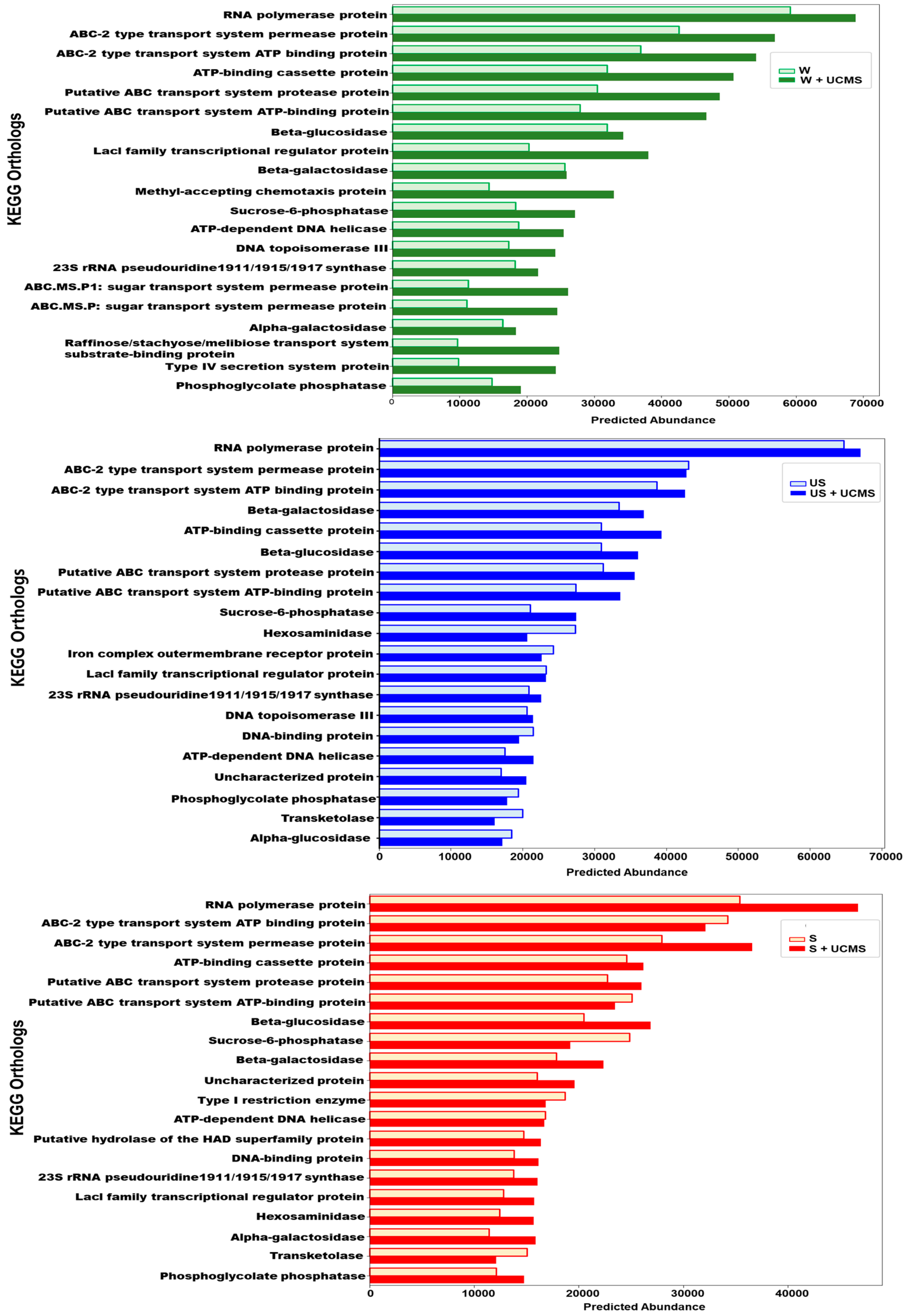

Figure S3. Alterations in gut microbiome profile

(A) Relative taxonomic abundance at the family levels and (B) Predicted Top 20 selected KEGG functional pathways. W, water; US, sucralose; S, sucrose; UCMS, unpredictable chronic mild stress.

.
